# Engineering Lipophilic Aggregation of Adapalene and Adamantane-Based Cocrystals via van der Waals Forces and Hydrogen Bonding

**DOI:** 10.1021/acs.cgd.4c00457

**Published:** 2024-05-30

**Authors:** Josephine Bicknell, Sidhaesh A. Agarwal, Kyle J. Petersen, Jesus Daniel Loya, Nicholas Lutz, Paulina M. Sittinger, Simon J. Teat, Nicholas S. Settineri, Gonzalo Campillo-Alvarado

**Affiliations:** † Department of Chemistry, Reed College, Portland, Oregon 97202-8199, United States; ‡ Institut für Chemie und Biochemie, Freie Universität Berlin, Arnimallee 22, 14195 Berlin, Germany; § Advanced Light Source, Lawrence Berkeley National Laboratory, Berkeley, California 94720, United States; ∥ Department of Chemistry, University of California, Berkeley, Berkeley, California 94720-1460, United States

## Abstract

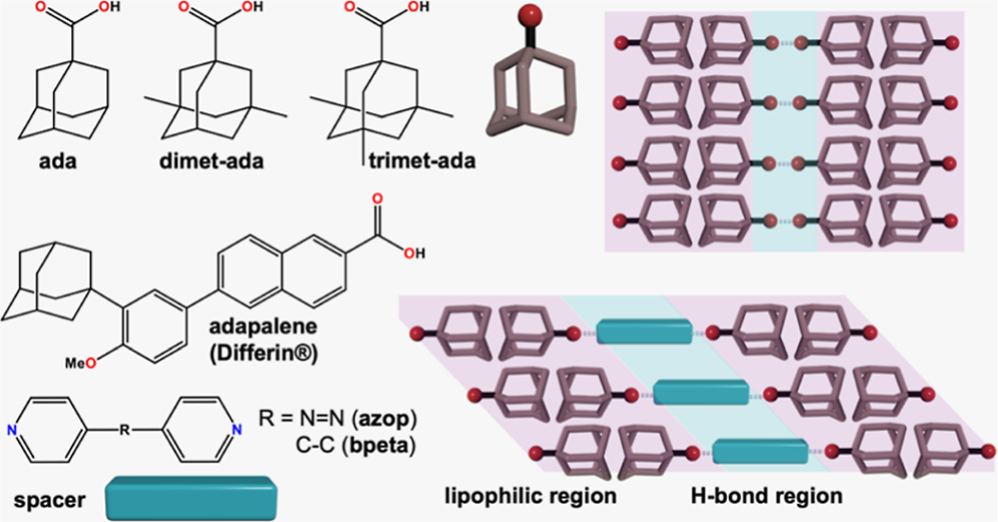

Lipophilic aggregation using adamantanes is a widely exploited molecular property in medicinal and materials chemistry. Adamantanes are traditionally installed to molecular units via covalent bonds. However, the noncovalent installation of adamantanes has been relatively underexplored and presents the potential to bring properties associated with adamantanes to molecules without affecting their intrinsic properties (e.g., pharmacophores). Here, we systematically study a series of adamantanecarboxylic acids with varying substitution levels of methyl groups and their cocrystals with bipyridines. Specifically, single-crystal X-ray diffraction shows that while the directionality of single-component adamantanes is notably sensitive to changes in methyl substitution, hydrogen-bonded cocrystals with bipyridines show consistent and robust packing due to π-stacking predominance. Our observations are supported by Hirshfeld surface and energy framework analyses. The applicability of cocrystal formation of adamantanes bearing carboxylic acids was used to generate the first cocrystals of adapalene, an adamantane-bearing retinoid used for treating acne vulgaris. We envisage our study to inspire noncovalent (i.e., cocrystal) installation of adamantanes to generate lipophilic aggregation in multicomponent systems.

## Abstract 2

A series of adamantanecarboxylic acids show profound changes in lipophilic aggregation upon methyl substitution, while their hydrogen-bonded cocrystal aggregates with 4,4′,-azopyridine exhibit tolerant and robust aggregation via π···π interactions. The applicability of cocrystal engineering of adamantanecarboxylic acids allowed the generation of the first cocrystals of adapalene, an adamantane-bearing retinoid with bipyridines.

## 1 Introduction

Adamantanes are emerging building blocks for active pharmaceutical ingredients (APIs) that treat diseases ranging from viral infections (influenza A) to neurodegenerative disorders (Parkinson’s disease).^1^ The effectiveness and medicinal properties of adamantane-based APIs rely on the cage-like hydrocarbon structure (“lipophilic bullet”),^2^ which enhances drugs’ lipophilicity,^3^ stability,^4^ and pharmacokinetics.^2^ Specifically, molecular dynamics simulations have demonstrated that adamantanes can be effectively introduced into the lipophilic part of lipid bilayers in membranes, which makes them desirable “add-ons” to pharmaceuticals.^5^ While strategies to modulate physicochemical properties using adamantane subunits typically rely on covalent bonding,^2,6^ reports of noncovalent (i.e., cocrystals)^7^ installation of adamantanes have been relatively scarce.^8^ A recent example by Katagiri et al. shows the potential of fine-tuning directionality of adamantane assemblies to form functional spherical aggregates with nanocavities up to ∼3 nm using a noncovalent approach. The cavities confine guests of different sizes (e.g., adamantanes, fullerenes, and metal–organic polyhedra).^9^ The work demonstrates that adamantanes can be powerful building blocks and design elements to generate functional materials by driving the directionality of the assembly process via lipophilic (i.e., van der Waals) interactions and have potential to be incorporated in diverse applications (e.g., drug delivery systems, nanodevices, and molecular sponges).^10^

**Scheme 1:**
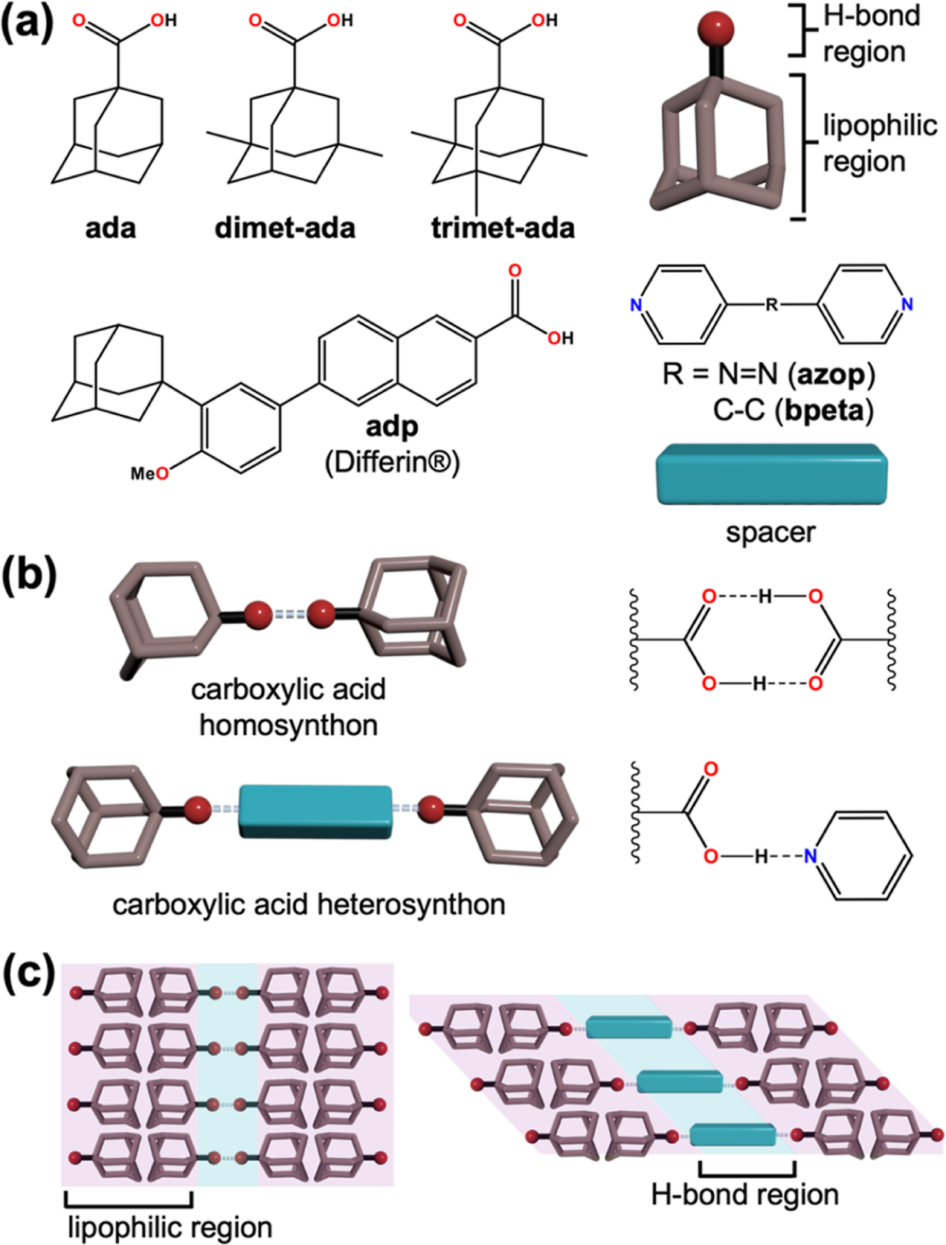
Supramolecular Interactions in Methyl-Substituted Adamantanecarboxylic Acids and Their Cocrystals with Bipyridines: (a) Molecular Building Blocks Used in This Study, (b) Supramolecular Synthons Formed with Adamanatanecarboxylic Acids, (c) Lipophilic and Hydrogen Bonding Regions Formed in Supramolecular Systems

Inspired by this work, we sought to understand how systematic modification of the hydrophobic cage in adamantanes could result in changes to crystal packing in single and multicomponent organic solids (Scheme 1). As a program of study to understand the molecular packing of adamantanes, we selected a series of adamantanecarboxylic acids with different substitution numbers of methyl groups: 1-adamantanecarboxylic acid (**ada**), 3,5-dimethyladamantane-1-carboxylic acid (**dimet-ada**), and 3,5,7-trimethyl-1-adamantanecarboxylic acid (**trimet-ada**) (Scheme 1a). Single-crystal X-ray diffraction (SCXRD) studies revealed the supramolecular architectures of the adamantanes to form dimers (i.e., homosynthons) via hydrogen bonds and also being strongly sensitive to the substituents in the adamantyl cage, generating supramolecular tapes, grids, and zigzag tapes, for **ada**, **dimet-ada**, and **trimet-ada**, respectively. To understand the effect of substitution in multicomponent supramolecular cocrystal aggregates, we selected 4,4′-azopyridine (**azop**) as a coformer for the adamantanecarboxylic acids. Cocrystals 2(**ada**)·(**azop**), 2(**dimet-ada**)·(**azop**), 2(**trimet-ada**)·(**azop**) form three-component assemblies via hydrogen bonds (i.e., heterosynthon) that organize into sheets (Scheme 1b). Although π-stacking of bipyridyl spacers provides consistent packing, the adamantane ends interact with varying degrees of packing efficiency (i.e., presence of voids) that result in side-by-side levels or interdigitated sheets (Scheme 1c). Our study is supported by Hirshfeld and energy framework analyses to account for the contributions of the supramolecular interactions in the crystal packing of cocrystals.

Applicability of the cocrystallization approach was extended to Adapalene (**adp**, brand name: Differin), an FDA-approved topical retinoid containing an adamantane bearing a carboxylic acid group, used for the treatment of acne vulgaris.^11^ Adapalene has a parabolic molecular structure that transfers into the curvature to cocrystal architectures with **azpop** and 1,2-bis(4-pyridyl)ethane (**bpeta**), aggregating via H-bonds and lipophilic interactions in cocrystals 2(**adp**)·(**azop**), and 2(**adp**)·(**bpeta**), respectively. To our knowledge, our work represents the first cocrystals of **adp** and adamantane-based drugs bearing a carboxylic acid motif. We envision that our contribution will inspire the installation of adamantanes via noncovalent interactions to generate robust and consistent supramolecular architectures driven by the combination of lipophilic aggregation with additional noncovalent interactions.

## 2 Experimental Section

### 2.1 Crystal and Cocrystal Synthesis

Methanol, benzene, ethyl acetate, and tetrahydrofuran (THF) were purchased from Sigma-Aldrich. Compounds **ada**, **dimet-ada**, **trimet-ada**, **adp**, **azop**, and **bpeta** were purchased from AmBeed, **trimet-ada** was purchased from Combi-Blocks. All chemicals were used as received without further purification. Crystals of **dimet-ada**, **trimet-ada** were afforded from ethyl acetate and methanol solutions (3 mL), respectively. Cocrystals of 2(**ada**)·(**azop**), 2(**dimet-ada**)·(**azop**), and 2(**trimet-ada**)·(**azop**) were generated by heat and sonication-assisted dissolution of the corresponding adamantane (0.36 mmol) and **azop** (0.18 mmol) in benzene (2.5 mL) and methanol (0.5 mL). 2**(adp)**·(**azop**) and 2**(adp)**·(**bpeta**) were generated by the dissolution of **adp** (0.15 mmol) and **azop** (0.08 mmol) or **bpeta** (0.08 mmol) in 2.5 mL of THF. Suitable single crystals for all samples formed via slow evaporation at room temperature approximately 1 week after preparation. Phase purity or composition was determined by analysis of powder X-ray diffraction (PXRD) data (see Supporting Information for additional experimental and PXRD data in Figures S13–S17).

### 2.2 X-ray Crystallography

SCXRD experiments for 2(**adp**)·(**azop**) and 2(**adp**)·(**bpeta**) were conducted either on a Bruker D8 diffractometer equipped with a PHOTON II CPAD detector with synchrotron radiation (λ = 0.7288 Å) on beamline 12.2.1 at Advanced Light Source or using a Rigaku XtaLAB Mini II diffractometer with a CCD area detector (λ = 0.71073 Å, graphite monochromator) for **dimet-ada**, **trimet-ada**, 2(**ada**)·(**azop**), 2(**dimet-ada**)·(**azop**), and 2(**trimet-ada**)·(**azop**). The intensity data with synchrotron radiation were collected in APEX3,^12^ integration and corrections were applied with SAINT v8.40a,^13^ and absorption and other corrections were made using SADABS 2016/2^14^ for 2(**adp**)·(**azop**) and TWINABS 2012/1 2(**adp**)·(**bpeta**). Dispersion corrections appropriate for this wavelength were calculated using the Brennan method in XDISP^15^ within WinGX.^16^ The structures were solved with a dual space method with SHELXT 2018/2^17^ and refined using SHELXL 2019/2.^18^ All non-hydrogen atoms were refined anisotropically. Hydrogen atoms were placed geometrically on the carbon atoms and refined with a riding model. On the H–O groups, they were found in the difference map and allowed to refine freely. Displacement parameter restraints were used to model C11′ more reasonably for 2(**adp**)·(**azop**). Collected data on the Rigaku XtaLAB Mini II diffractometer underwent standard data reduction and background correction from the integrated CrysAlisPro package. Structural refinement and solution were performed with Olex2,^19^ SHELXL,^18^ and SHELXT.^17^ Crystallographic data and selected metrics for the starting material and cocrystal structures are summarized in Tables S1–S9 (see Supporting Information). PXRD data were collected on a Scintag XDS-2000 diffractometer using CuKα1 radiation (λ = 1.5418 Å). The samples were mounted and collected on glass slides typically in the range of 5–40° two-theta (scan type: step size: 0.02°, rate: 3 deg/min, continuous scan mode). The equipment was operated at 40 kV and 30 mA, and data were collected at room temperature. Fourier-transform infrared (FT-IR) spectroscopy spectra were captured using a Thermo Fischer Scientific iS5 IR spectrometer from 600 to 4000 cm^–1^ using a diamond attenuated total reflectance (ATR) accessory.

## 3 Results and Discussion

### 3.1 Rationale toward Design

Adamantanes naturally occur in petroleum and are chemically manufactured in high yields via superacid catalysis.^20^ The caged structures present opportunities in crystal engineering as lipophilic building blocks, serving as “sticky nodes” during self-assembly in single and multicomponent systems using stabilizing van der Waals interactions (i.e., London dispersion forces).^21^ The molecular structure of adamantanes in the solid state has also resulted in macroscopic material properties such as plasticity (i.e., disorder–order phase transitions) and^22^ flexibility (i.e., fibrous morphologies),^23^ which has increased their interest as a functional building block. Although individual van der Waals forces are regularly considered weak, cooperative and London dispersion forces^21^ have been demonstrated to be strong enough to stabilize and elongate carbon–carbon bonds up to 1.67 Å in sterically hindered diamondoid dimers.^24^ We envisage van der Waals forces can also provide robust structural junctions to supramolecular systems.

To understand the consistency and robustness of lipophilic aggregation in adamantanes bearing a carboxylic acid, we crystallized **dimet-ada** and **trimet-ada**, which are bulky, sterically congested sp^3^-rich cages, to inspect their crystal structures using SCXRD. Reported plain adamantane structure **ada** (CSD refcode: VIDSIK)^25^ was used for comparison. Energy framework calculations were performed using CrystalExplorer with the Hartree–Fock model with the 3-21G basis set.^26^

**Figure 1:**
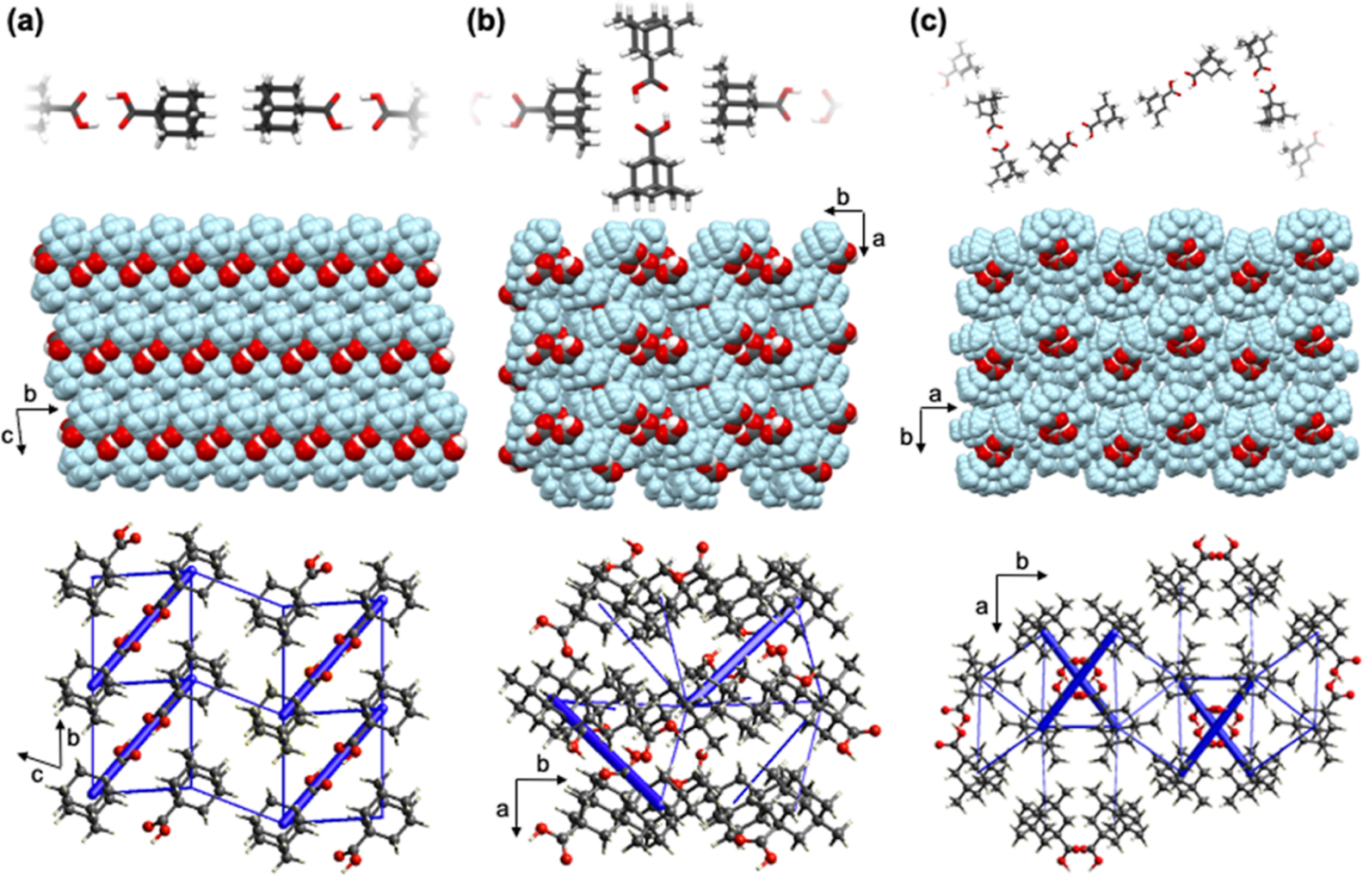
X-ray crystal structures showing short contacts between adamantane cages (top), spacefill view of extended packing (middle), and energy frameworks (total energy) (bottom) of (a) **ada**, (b) **dimet-ada**, and (c) **trimet-ada**.

SCXRD studies revealed the structures of **ada**, **dimet-ada**, and **trimet-ada** as pure forms to form dimers via [O–H···O] hydrogen bonds (homosynthons). Adamantanecarboxylic acid homodimers are consistent with previously reported systems.^25,27^ The carboxylic acid dimers show notable differences in the extended packing due to the surface morphologies of their hydrocarbon cages, generating different [H···H] contact patterns (London dispersion forces). In all the systems, hydrogen bonding has the highest energetic contributions to energy frameworks (i.e., the thickest tubes show the strongest stabilizing energetic contributions from Coulombic interactions), while dispersion interactions act as supplementary energy contributors that support the supramolecular fabric (Figure 1).

The unsubstituted structure **ada** crystallizes in the triclinic space group *P*-1. The **ada** molecules organize as tapes oblique to the *ac*-axes. The tapes are slightly corrugated by 1.53 Å (interplanar distance) as measured by parallel planes measured by oxygen atoms in dimers. Extended packing shows the presence of adamantane cage bilayers that run along the *b*-axis and are supported by additional [H···H] contacts (Figure 1a).

The components of **dimet-ada** crystallize in the monoclinic space group *P*2_1_. In the structure, dimers are oriented 24.1° from each other, as measured by parallel planes measured by oxygen atoms, resulting in a supramolecular grid. Additional [C–H···O] contacts support the orthogonal orientation of dimethyladamantane cages in relation to the carboxylic acid groups, which effectively form a hydrophilic pocket (Figure 1b).

Trisubstituted **trimet-ada** crystallizes in the monoclinic space group *P*2_1_/*c*. The dimeric assemblies of adamantanecarboxylic acid dimers organize as zigzag tapes showing [H···H] contacts that direct the assembly morphologically. The best places from adjacent dimers in the zigzag tapes have a fold angle 30.1° and interact with adjacent tapes via additional [H···H] contacts, which effectively envelop the carboxylic acid motifs in hydrophilic pockets (Figure 1c).

Isodensity surfaces (0.002 au) calculated in Spartan 20 from SCXRD coordinates show changes in geometries of the hydrocarbon cages by the installation of methyl groups and subsequent volume increments, ranging from 183 to 423.6 Å^3^ in **ada** and **adp**, respectively (Figure S12, see Supporting Information). Despite the notable changes in supramolecular architectures and space groups, the hydrocarbon cages showed no significant difference in contributions from [H···H] contacts, as measured by Hirshfeld surface analysis maps (Figure S10, see Supporting Information).

### 3.2 X-ray Structures of Adamantane Cocrystals

To understand the organizing role of adamantanes in multicomponent systems, we cocrystallized adamantanes with **azop**, which acted as a supramolecular bridge via [O–H···N] hydrogen bonds in all systems.

#### 3.2.1 Cocrystal 2(**ada**)·(**azop**)

**Figure 2:**
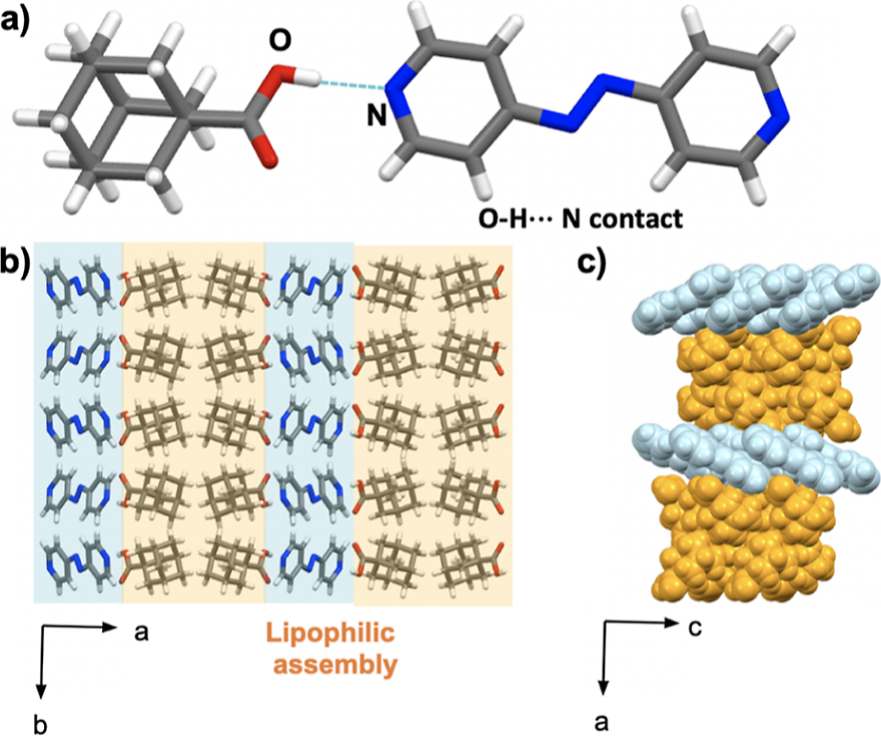
X-ray crystal structure of 2(**ada**)·(**azop**): (a) molecular units sustained by [O–H···N] hydrogen bonds, (b) lipophilic assemblies of cocrystal aggregates in the *ab*-plane, and (c) spacefill view of level sheets along the *c*-axis.

The components in 2(**ada**)·(**azop**) crystallize in the orthorhombic space group *Pccn* with an asymmetric unit that contains one **ada** unit linked with one-half of the **azop** spacer. The extended structure shows three-component assemblies sustained by [O–H···N] hydrogen bonds (1.87 Å) (Figure 2a). Pyridyl rings in adjacent assemblies exhibit face-to-face [π···π] contacts (3.72 Å), which support the formation of sheets. Dispersion forces in van der Waals [H···H] contacts produce a lipophilic aggregation of the adamantyl motifs (Figure 2b). The aggregation is further supported by [C–H···O] contacts. Extended view of the supramolecular aggregate shows the solid to be close-packed with no voids present. Weak [C–H···N] hydrogen bonds (2.06 Å) occur between **ada** and **azop** components and further organize the assembly into level sheets along the *c*-axis (Figure 2c). FT-IR spectroscopy showed the formation of bands around 2505 cm^–1^ (acid-pyridine O–H stretch) and 1892 cm^–1^ (acid-pyridine H-bonded O–H stretch), which are characteristic of an acid–pyridine synthon in cocrystals (Figure S18, see Supporting Information).^28^

#### 3.2.2 Cocrystal 2(**dimet-ada**)·(**azop**)

**Figure 3:**
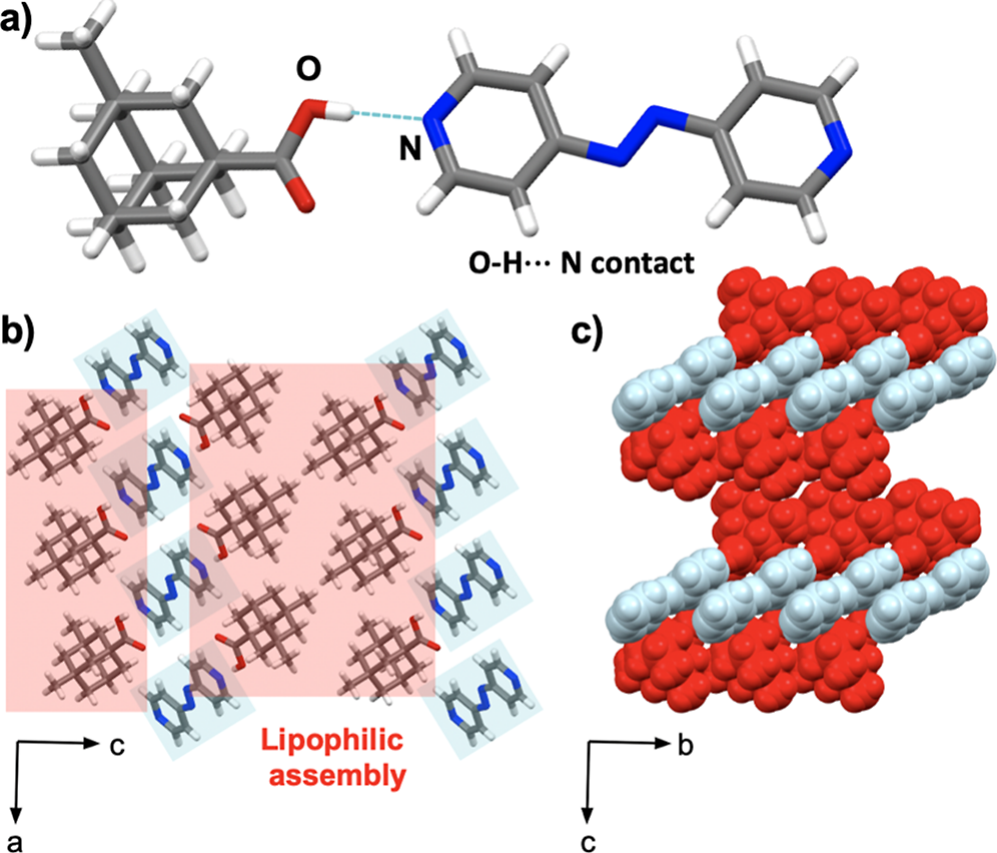
X-ray crystal structure of 2(**dimet-ada**)·(**azop**): (a) molecular units sustained by [O–H···N] hydrogen bonds, (b) lipophilic assemblies of cocrystal aggregates in the *ac*-plane, and (c) spacefill view of corrugated sheets along the *b*-axis.

The compounds of 2(**dimet-ada**)·(**azop**) crystallize in the triclinic space group *P*1. As with 2(**ada**)·(**azop**), the asymmetric unit contains one **dimet-ada** unit linked with the **azop** spacer via [O–H···N] hydrogen bonds (1.90 Å) (Figure 3a), forming a three-component assembly. The components assemble in lipophilic aggregates sustained by face-to-face [π···π] contacts (3.60 Å) between adjacent pyridyl spacers and van der Waals [H···H] contacts (Figure 3b). In contrast to 2(**ada**)·(**azop**), the interactions organize the aggregates into corrugated sheets along the *b*-axis (Figure 3c). FT-IR spectroscopy showed the formation of acid–pyridine synthon bands around 2485 cm^–1^ (acid-pyridine O–H stretch) and 1880 cm^–1^ (acid-pyridine H-bonded O–H stretch), as observed in the 2(**ada**)·(**azop**) cocrystal (Figure S19, see Supporting Information).

#### 3.2.3 Cocrystal 2(**trimet-ada**)·(**azop**)

**Figure 4:**
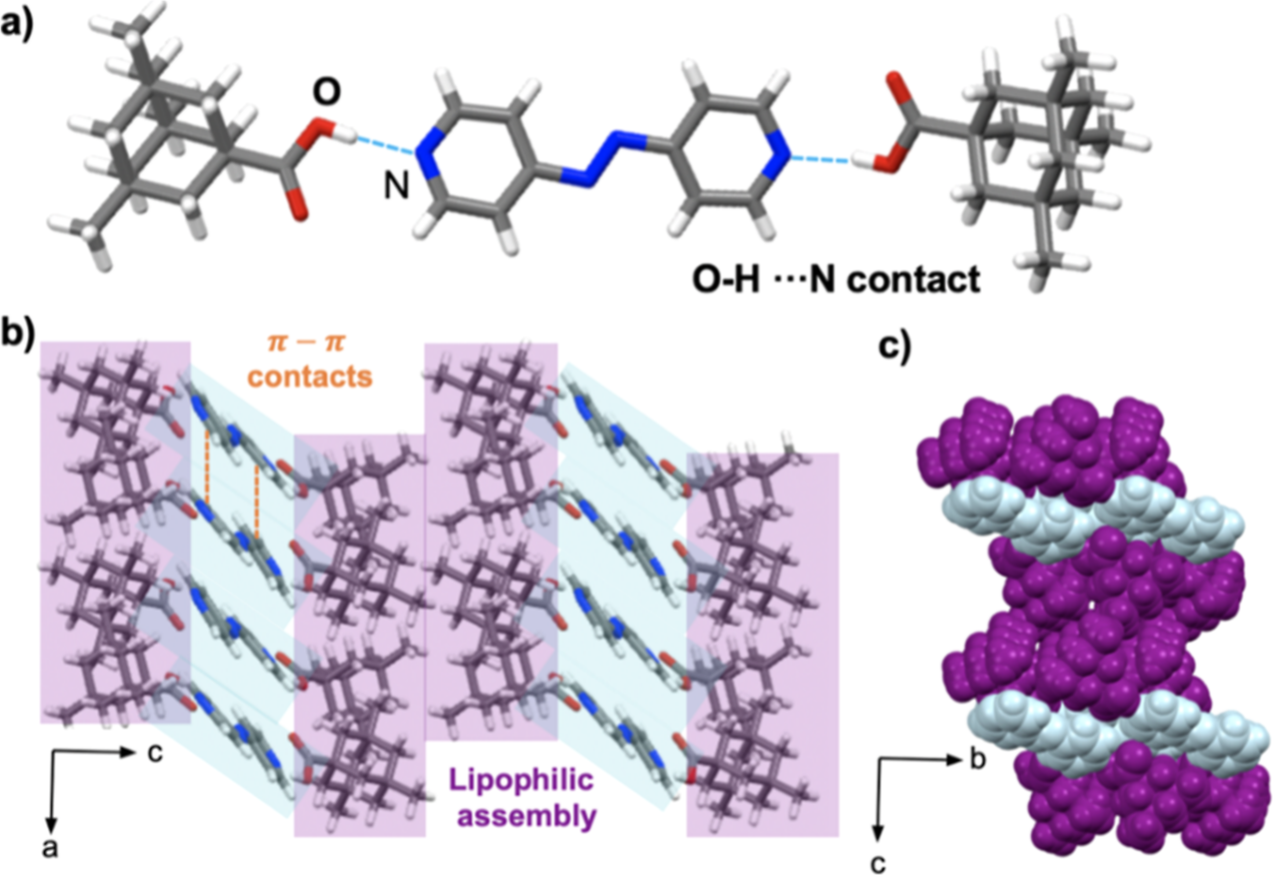
X-ray crystal structure of 2(**trimet-ada**)·(**azop**): (a) molecular units sustained by [O–H···N] hydrogen bonds, (b) lipophilic assemblies of cocrystal aggregates in the *ac*-plane, and (c) spacefill view of corrugated sheets along the *b*-axis.

The compounds of 2(**trimet-ada**)·(**azop**) crystallize in the triclinic space group *P*1 to form three-component assemblies that are sustained by [O–H···N] hydrogen bonds (1.88 and 1.87 Å) (Figure 4a). As shown in 2(**ada**)·(**azop**) and 2(**dimet-ada**)·(**azop**) cocrystals, the **azop** molecule shows consistent [π···π] contacts (3.56 Å) between adjacent spacers, which drive the lipophilic aggregation via [H···H] van der Waals contacts (Figure 4b). The extended view of the supramolecular aggregate shows the solid architecture to be further sustained by [C–H···O] hydrogen bonds (2.89 Å) between a methyl group in the adamantyl ring and the carboxylic acid from an adjacent unit. Additional [C–H···O] hydrogen bonds between the pyridyl ring and an adjacent adamantyl carboxylic acid (2.88 Å) organize the aggregate into corrugated sheets along the *b*-axis (Figure 4c). Formation of the acid–pyridine synthon was confirmed by FT-IR spectroscopy with bands around 2499 cm^–1^ (acid-pyridine O–H stretch) and 1911 cm^–1^ (acid-pyridine H-bonded O–H stretch) (Figure S19, see Supporting Information).

### 3.3 X-ray Structures of Adapalene Cocrystals

Adapalene (**adp**) is an adamantane-bearing retinoid with a parabolic architecture. Cocrystals of **adp** with **azop** and **bpeta** were grown from a THF solution as orange and colorless planks, respectively.

**Figure 5:**
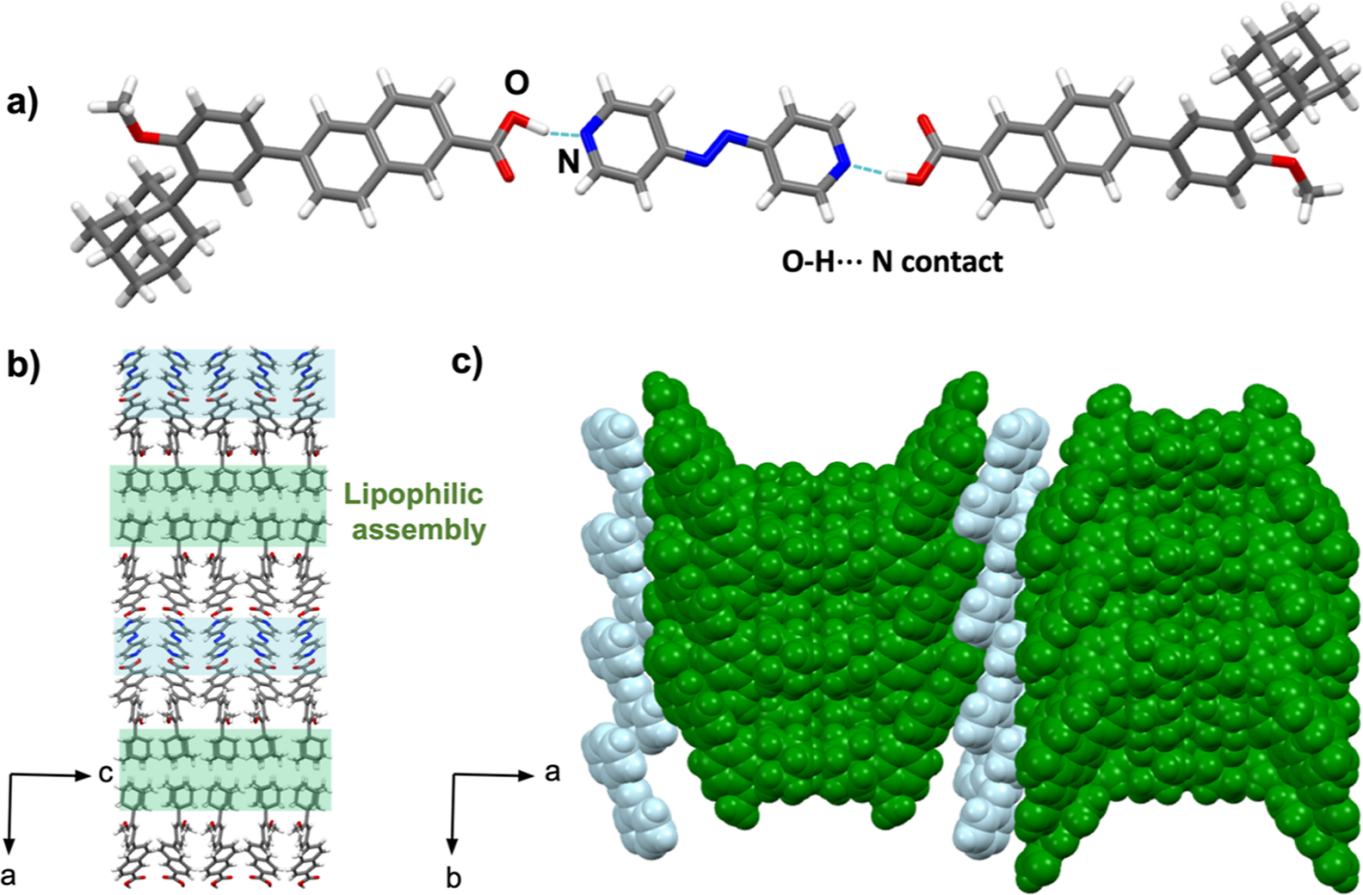
X-ray crystal structure of 2(**adp**)·(**azop**): (a) molecular units sustained by [O–H···N] hydrogen bonds, (b) lipophilic assemblies of cocrystal aggregates in the *ac*-plane, and (c) spacefill view of sinusoidal sheets along the *a*-axis.

Cocrystal 2(**adp**)·(**azop**) crystallizes in the orthorhombic *Pca*2_1_ space group with an asymmetric unit that contains two **adp** units linked with the **azop** molecule via [O–H···N] hydrogen bonds (1.53 Å) (Figure 5a). The three-component assembly is further sustained by [C–H···O] hydrogen bonds involving **adp** and **azop** and **adp** from adjacent assemblies. The shorter [O–H···N] hydrogen bond results in a tighter lipophilic assembly in comparison to the previous adamantyl cocrystals. The parabolic architecture is supported by the side-by-side arrangement of adamantyl supported by [C–H···π] contacts (Figure 5b). The pyridyl rings exhibit face-to-face [π···π] stacking adjacent **azop** molecules (3.92 Å) and naphthyl rings from **adp** rings (3.76 Å). Together, the interactions organize the aggregate into sinusoidal sheets along the *a*-axis (Figure 5c). Consistent with the previous cocrystals of adamantanecarboxylic acids with **azop**, FT-IR spectroscopy of 2(**adp**)·(**azop**) showed the appearance of bands around 2423 and 1919 cm^–1^, which are consistent with the formation of acid-pyridine O–H stretch and acid-pyridine H-bonded O–H stretch, respectively (Figure S20, see Supporting Information).

**Figure 6:**
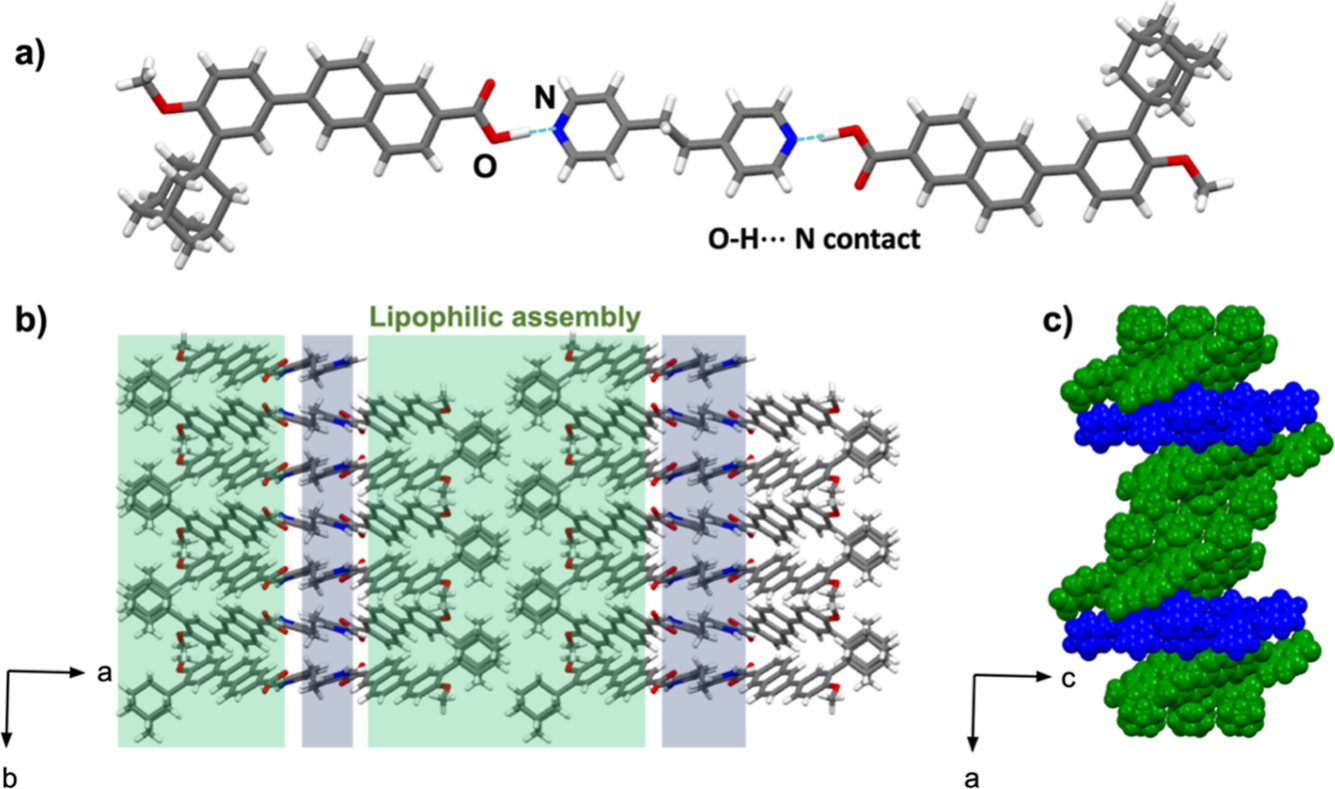
X-ray crystal structure of 2(**adp**)·(**bpeta**): (a) molecular units sustained by [O–H···N] hydrogen bonds, (b) corrugated lipophilic assemblies in the *ab*-plane, and (c) spacefill view of corrugated sheets along the *c*-axis.

The compounds 2(**adp**)·(**bpeta**) crystallize in a monoclinic *P*2_1_/*c* space group with an asymmetric unit that comprises one **adp** unit and one-half of **bpeta** unit linked by a short [O–H···N] hydrogen bond (1.29 Å) (Figure 6a). The close-packed structure is organized into a lipophilic assembly, however, while 2(**adp**)·(**azop**) showed side-by-side packing of the adamantyl motifs (Figure 5b), the adamantyl groups in 2(**adp**)·(**bpeta**) show an interlocking pattern (Figure 6b, interlocking pattern highlighted in green). The pyridyl rings within this assembly exhibit edge-to-face [π···π] contacts between **bpeta** and the naphthyl ring of **adp** rings rather than face-to-face stacking as in 2(**adp**)·(**azop**). The edge-to-face arrangement is supported by [C–H···O] and [C–H···N] hydrogen bonds between a methylene group of **bpeta** and naphthyl group of **adp**. The pattern in the [π···π] contacts (e.g., edge-to-face vs face-to-face) between the 2(**adp**)·(**azop**) and 2(**adp**)·(**bpeta**) structure results in a distinct supramolecular organization. Without the two face-to-face [π···π] contacts, the 2(**adp**)·(**bpeta**) structure does not adopt the sinusoidal formation of 2(**adp**)·(**azop**), and instead resembles the corrugated organization observed in 2(**dimet-ada**)·(**azop**) along the *c*-axis (Figure 6c). FT-IR spectrum of 2(**adp**)·(**bpeta**) is comparable with the one for 2(**adp**)·(**azop**) as shown in the appearance of bands for the acid–pyridine synthon around 2458 and 1936 cm^–1^ (Figure S22, see Supporting Information).

### 3.4 Utility of Adamantanes for Crystal Engineering

**Figure 7:**
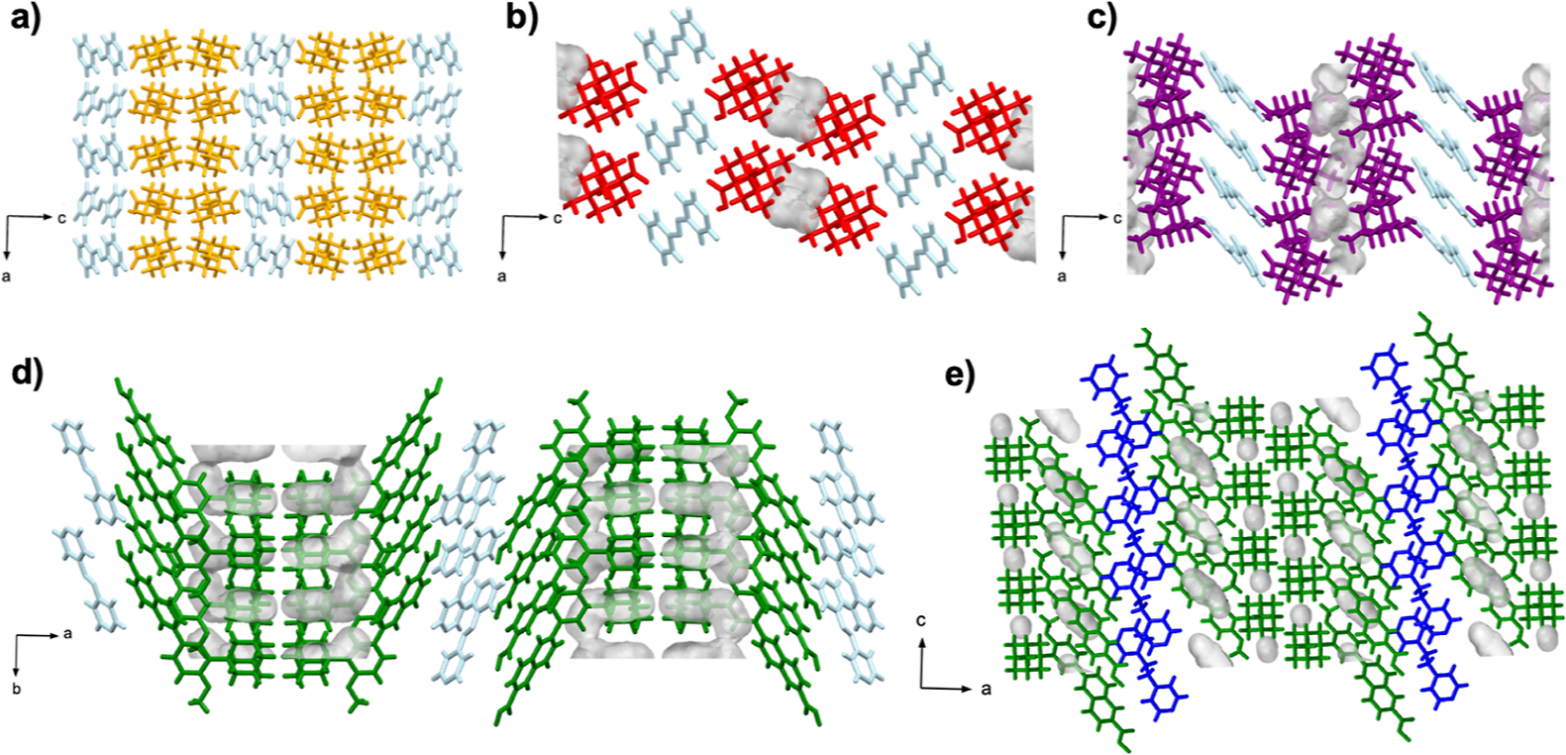
Void analysis of X-ray crystal structures of cocrystals: (a) 2(**ada**)·(**azop**), (b) 2(**dimet-ada**)·(**azop**), (c) 2(**trimet-ada**)·(**azop**), (d) 2(**adp**)·(**azop**), and (e) 2(**adp**)·(**bpeta**).

Our observations highlight the propensity of adamantanecarbocylic acids and the corresponding hydrogen-bonded cocrystals with **azop** and **bpeta** to form aggregates sustained by van der Waals [H···H] contacts. Lipophilic aggregation in adamantanecarboxylic acid cocrystals with **azop** to be consistent and tolerant, akin to long-range synthon Aufbau modules (LSAMs) introduced by Ganguly and Desiraju. LSAMs,^7c,29^ or long-range synthons, can influence the crystal packing of organic solids by transferring known large synthons (i.e., adamantane aggregation) into derived solids. Despite the similar packing arrangement of cocrystals, which is primarily driven by [O–H···N] hydrogen bonds and [π···π] contacts, we identified the presence of voids in methylated structures 2(**dimet-ada**)·(**azop**) and 2(**timet-ada**)·(**azop**), which indicates a frustrated packing (Figure 7).^30^ Specifically, voids occupying 4.7, and 5.6% of total unit cell volumes were found in crystal structures of 2(**dimet-ada**)·(**azop**), and 2(**timet-ada**)·(**azop**), respectively. In contrast, nonsubstituted 2(**ada**)·(**azop**) did not show the presence of voids (Table 1). Frustrated systems are a suitable platform to engineer supramolecular systems for confinement (e.g., solvates, hydrates), and the formation of additional multicomponent solids.^10f,31^ Differences in packing are also supported by Hirshfeld surface analysis (see Supporting Information), which show van der Waals [H···H] contacts to be the main contributors in the crystal packing. The addition of methyl groups corresponds to an enrichment of [H···H] contacts as observed in a previous study.^32^

**Table 1:** Selected Metrics of Adamantane-Based Cocrystals 2(**ada**)·(**azop**), 2(**dimet-ada**)·(**azop**), 2(**trimet-ada**)·(**azop**), 2(**adp**)·(**azop**), and 2(**adp**)·(**bpeta**)

cocrystal	d[O–H···N] (Å)	d[π···π] (Å)	void (%)^c^	[H···H] contribution (%)^d^
2(**ada**)·(**azop**)	1.87	3.72^a^	0	61.8
2(**dimet-ada)**·**(azop**)	1.90	3.60^a^	4.7	66.8
2(**trimet-ada)**·**(azop**)	1.88, 1.87	3.56^a^	5.6	72.0
2(**adp**)·(**azop**)	1.53	3.92,^a^ 3.76^b^	7.9	59.9
2(**adp**)·(**bpeta**)	1.29	NA^e^	5.0	55.4

a Measured using calculated centroids in pyridyl rings.

b Measured using calculated centroids in pyridyl and naphthyl rings.

c Calculated using 1 Å probe radius, 0.3 Å grid spacing.

d Contributions obtained from Hirshfeld surface analysis of crystal packing out of total surface.

e Edge-to-face π-stacking.

## 4 Conclusions

We have systematically evaluated the ability of adamantanecarboxylic acids with varying levels of substitution with methyl groups to form lipophilic aggregates in single- and multicomponent crystals. Specifically, crystal packing of single component adamantanes exhibited significant changes in directionality of dimers by the addition of methyl groups, while multicomponent cocrystal aggregates were more tolerant to supramolecular changes. Specifically, cocrystal aggregates showed consistent packing primarily dictated by [O–H···N] hydrogen bonds and [π···π] contacts, while van der Waals forces acted as ancillary binding forces with minor supramolecular modifications throughout the aggregate series. The cocrystal aggregate strategy of adamantanecarboxylic acids was utilized to form the first cocrystals of **adp**, an adamantane-containing retinoid, with **azop** and **bpeta**. The **adp** cocrystals showed sinusoidal and corrugated sheets that were responsive to the interactions from the parabolic **adp** architecture with **azop** and **bpeta**, respectively. Given the wide commercial availability and relevance of adamantanes for medicinal chemistry^2^ and materials science,^10e^ we are now exploring the uses of additional adamantane molecules to support the formation of frustrated solids to function as molecular hosts.

## Supporting Information Available

X-ray crystallographic information files (CIF) are available for compounds **dimet-ada**, **trimet-ada**, **2(ada)**·**(azop)**, **2(dimet-ada)**·**(azop)**, **2(trimet-ada)**·**(azop)**, **2(adp)**·**(azop)**, **2(adp)**·**(bpeta)** (CCDC accession codes: 2343560–2343566). Experimental conditions, FT-IR spectra, PXRD patterns, and additional SCXRD data (PDF)
