# Supplementary material for: Engineering Lipophilic Aggregation of Adapalene and Adamantane-Based Cocrystals via van der Waals Forces and Hydrogen Bonding
Source: Cryst Growth Des. 2024 May 30;24(12):5222–30. doi: 10.1021/acs.cgd.4c00457 (PMC11191584; doi:10.1021/acs.cgd.4c00457)
Supplement: Supplementary file 1 — cg4c00457_si_001.pdf [file cg4c00457_si_001.pdf]

Supporting Information (SI)

Engineering Lipophilic Aggregation of Adapalene and Adamantane-based Cocrystals via van der Waals Forces and Hydrogen Bonding

Josephine Bicknell,[a] Sidhaesh A. Agarwal,[a] Kyle J. Petersen,[a] Jesus Daniel Loya,[a] Nicholas Lutz,[a] Paulina M. Sittinger,[a,b] Simon J. Teat,[c] Nicholas S. Settineri,[c,d] and Gonzalo Campillo-Alvarado[a,*]

[a] Department of Chemistry, Reed College, Portland, OR 97202-8199, USA, [b] Institut für Chemie und Biochemie, Freie Universität Berlin, Arnimallee 22, 14195 Berlin, Germany, [c] Advanced Light Source, Lawrence Berkeley National Laboratory, Berkeley, CA 94720, USA, [d] Department of Chemistry, University of California, Berkeley, Berkeley, California 94720-1460, United States.

*e-mail: gcampillo@reed.edu

Table of Contents:

S1) Experimental information

S2) Single-crystal X-ray data

S3) Powder X-ray data

S4) FT-IR data

S5) NMR spectral data

S1. Experimental information

Materials:

Methanol, benzene, ethyl acetate, and tetrahydrofuran (THF) were purchased from Sigma-Aldrich. Compounds 1-adamantanecarboxylic acid (**ada**), 3,5-dimethyladamantane-1-carboxylic acid (**dimet-ada**), adapalene (**adp**), 4,4'-azopyridine (**azop**) and 1,2-bis(4-pyridyl)ethane (**bpeta**) were purchased from AmBeed, and 3,5,7-trimethyl-1-adamantanecarboxylic acid (**trimet-ada**) was purchased from Combi-Blocks. All chemicals were used as received without further purification.

Crystallization of **dimet-ada**: 3,5-dimethyladamantane-1-carboxylic acid (**dimet-ada,** 30 mg, 0.14 mmol**)** was dissolved in 3mL of ethyl acetate. The solution was left for one week of slow crystallization and suitable crystals for single crystal X-ray diffraction (SCXRD) were collected.

Crystallization of **trimet-ada**: 3,5,7-trimethyl-1-adamantanecarboxylic acid (**trimet-ada,** 30 mg, 0.13 mmol) was dissolved in 3 mL of methanol. The solution was left for one week of slow crystallization and suitable crystals for SCXRD were collected.

Synthesis of 2(**ada**)·(**azop**): 1-adamantanecarboxylic acid (**ada**, 64.9 mg, 0.36 mmol) and 4,4'-azopyridine (**azop**, 33.2 mg, 0.18 mmol) were dissolved in 2.5 mL of benzene with methanol added dropwise until dissolved. The sample was heated and stirred between additions of methanol until the solution was completely transparent. The solution was left for one week of slow crystallization, and orange irregular crystals suitable for SCXRD were collected.

Synthesis of 2(**dimet-ada**)·(**azop**): 3,5-dimethyladamantane-1-carboxylic acid (**dimet-ada,** 75.0 mg, 0.36 mmol) and 4,4'-azopyridine (**azop**, 33.2 mg, 0.18 mmol) were dissolved in 2.5 mL of benzene with methanol added dropwise until dissolved. The sample was heated and stirred between additions of methanol until the solution was completely transparent. The solution was left for one week of slow crystallization, and red block-like crystals suitable for SCXRD were collected.

Synthesis of 2(trimet-ada)·(azop):3,5,7-trimethyl-1-adamantanecarboxylic acid (80.0 mg, 0.36 mmol) and 4,4'-azopyridine (**azop**, 33.2 mg, 0.18 mmol) were dissolved in 2.5 mL of benzene with methanol added dropwise until dissolved. The sample was heated and stirred between additions of methanol until the solution was completely transparent. The solution was left for one week of slow crystallization, and red plate-like crystals suitable for SCXRD were collected.

Synthesis of 2(adp)·(azop): Adapalene (Differin®) (**adp,** 61.9 mg, 0.15 mmol) and 4,4'-azopyridine (**azop**, 14.7 mg, 0.08 mmol) were dissolved in 2.5 mL of THF. The solution was left for one week of slow crystallization, and orange plate-like crystals suitable for synchrotron were collected.

Synthesis of 2(adp)·(bpeta) Adapalene (Differin®) (**adp,** 61.9 mg, 0.15 mmol) and 1,2-bis(4-pyridyl)ethane (**bpeta,** 14.7 mg, 0.08 mmol) were dissolved were dissolved in 2.5 mL of THF. The solution was left for one week of slow crystallization, and colorless plate-like crystals suitable for the synchrotron were collected.

Instruments and methods:
Single crystal X-ray diffraction (SCXRD) data was collected on a Rigaku XtaLAB Mini II diffractometer with a CCD area detector ($\lambda MoK\alpha$ = 0.71073 Å, monochromator: graphite) equipped with an Oxford Cryostream low-temperature device. Experiments were conducted at 100 K with a range of 2θ = 3-62°. The collected data was refined with CrysAlisPro through standard data reduction and background corrections (multiscan for **ada, dimet-ada**, **trimet-ada**, 2(**ada**)·(**azop**), 2(**dimet-ada**)·(**azop**), 2(**trimet-ada**)·(**azop**)). Crystals were mounted in Paratone oil on a Mitegen magnetic mount. Structure solution and refinement were performed using SHELXT[1] and SHELXL,[2] respectively, within the Olex2[3] graphical user interface. Non-hydrogen atoms were refined anisotropically, and hydrogen atoms were placed in geometrically calculated positions using a riding model. Synchrotron radiation was used to collect 2(**adp**)·(**azop**) and 2(**adp**)·(**bpeta**); The intensity data were collected with APEX3,[4] integration and corrections were applied with SAINT v8.40a,[5] absorption and other corrections were made using SADABS 2016/2[6] for 2(**adp**)·(**azop**) and TWINABS 2012/1 for 2(**adp**)·(**bpeta**). Dispersion corrections appropriate for this wavelength were calculated using the Brennan method in XDISP[7] within

WinGX.[8] The structures were solved with a dual space method with SHELXT 2018/2 and refined using SHELXL 2019/2. All non-hydrogen atoms were refined anisotropically. Hydrogen atoms were placed geometrically on the carbon atoms, refined with a riding model. On the H-O groups they were found in the difference map and allowed to refine freely. Crystal structures were generated using Mercury.

Additional refinement notes on 2(**adp**)·(**azop**): Displacement parameter restraints were used to model C11' more reasonably. All non-hydrogen atoms were refined anisotropically. Hydrogen atoms were placed geometrically on the carbon atoms, refined with a riding model. On the H-O groups they were found in the difference map and allowed to refine freely. Displacement parameter restraints were used to model C11' more reasonably. PLATON and CHECKCIF found 93% fit to *Pbca*. The refinement was tried in *Pbca* but the R1 stuck around 15% cf 7.1% in $Pca2_1$ and the refinement output did not flag any correlation in the refinement. Therefore, the structure was refined in $Pca2_1$.

Additional refinement notes on 2(**adp**)·(**bpeta**): Several crystals were tried, and this was the best crystal. The diffraction pattern showed twinning. Using Cell_now two orientation matrices were determined, the relationship between these components was determined to be 180 degrees about real axis 0 0 1. The data were integrated using the two matrices in SAINT, TWINABS was used to produce a merged HKLF4 file, for structure solution and initial refinement, and HKLF5 file for final structure refinement. The HKLF5 file contained the merged reflections first component and those that overlapped with this component, which were split into 2 reflections. TWINABS indicated the twin faction to be 64:36 The structure was solved using the HKLF4 file, but the best refinement was given by the HKLF5 file. All non-hydrogen atoms were refined anisotropically. Hydrogen atoms were placed geometrically on the carbon atoms, refined with a riding model. On the H-O groups they were found in the difference map and allowed to refine freely.

Powder X-ray diffraction (PXRD) data was collected on a Scintag XDS-2000 diffractometer using $CuK\alpha_1$ radiation (λ = 1.5418 Å). The samples were mounted and collected on glass slides typically in the range of 5−40° two-theta (scan type: step size: 0.02°, rate: 3 deg/min, continuous scan

mode). The equipment was operated at 40 kV and 30 mA, and data was collected at room temperature. Fourier-transform infrared spectroscopy (FT-IR) spectra were captured using a ThermoFischer Scientific iS5 IR spectrometer from 600 to 4000 cm-1 using a diamond attenuated total reflectance (ATR) accessory.

S2. Single-crystal X-ray data

Table S1. Crystallographic parameters for **dimet-ada**

**Compound name**	**dimet-ada**
**Empirical formula**	$C_{13}H_{20}O_2$
**Formula weight**	208.29
**Temperature/K**	99.9(6)
**Crystal system**	monoclinic
**Space group**	$P2_1$
**a/Å**	12.3815(4)
**b/Å**	15.2966(4)
**c/Å**	13.3061(4)
**α/°**	90
**β/°**	108.971(3)
**γ/°**	90
**Volume/Å^3**	2383.22(13)
**Z**	8
**ρ_{calc}g/cm^3**	1.161
**μ/mm^{-1}**	0.076
**F(000)**	912.0
**Crystal size/mm^3**	0.433 × 0.299 × 0.194
**Radiation**	Mo Kα (λ = 0.71073)
**2Θ range for data collection/°**	3.906 to 49.998
**Index ranges**	$-14 \leq h \leq 14$, $-17 \leq k \leq 18$, $-15 \leq l \leq 15$
**Reflections collected**	17622
**Independent reflections**	7921 [R_{int} = 0.0321, R_{sigma} = 0.0443]
**Data/restraints/parameters**	7921/1/553
**Goodness-of-fit on F^2**	0.988
**Final R indexes [I>=2σ (I)]**	R_1 = 0.0389, wR_2 = 0.0948
**Final R indexes [all data]**	R_1 = 0.0524, wR_2 = 0.1008
**CDCC Identification Code**	2343564

Table S2. Crystallographic parameters for **trimet-ada**

**Compound name**	**trimet-ada**
**Empirical formula**	$C_{28}H_{44}O_4$
**Formula weight**	444.63
**Temperature/K**	100.00(10)
**Crystal system**	monoclinic
**Space group**	$P2_1/c$
**a/Å**	15.0002(8)
**b/Å**	21.6526(10)
**c/Å**	7.8611(5)
**α/°**	90
**β/°**	98.494(6)
**γ/°**	90
**Volume/Å^3**	2525.2(2)
**Z**	4
**ρ_{calc}g/cm^3**	1.170
**μ/mm^{-1}**	0.076
**F(000)**	976.0
**Crystal size/mm^3**	0.587 × 0.126 × 0.071
**Radiation**	Mo Kα (λ = 0.71073)
**2Θ range for data collection/°**	4.658 to 49.996
**Index ranges**	$-17 \leq h \leq 17$, $-24 \leq k \leq 25$, $-9 \leq l \leq 8$
**Reflections collected**	19839
**Independent reflections**	4443 [R_{int} = 0.1764, R_{sigma} = 0.1068]
**Data/restraints/parameters**	4443/0/297
**Goodness-of-fit on F^2**	0.960
**Final R indexes [I>=2σ (I)]**	R_1 = 0.0719, wR_2 = 0.1784
**Final R indexes [all data]**	R_1 = 0.1094, wR_2 = 0.2053
**CDCC Identification Code**	2343562

Table S3. Crystallographic parameters for 2(**ada**)·(**azop**)

**Compound name**	2(**ada**)·(**azop**)
**Empirical formula**	$C_{16}H_{20}N_2O_2$
**Formula weight**	272.34
**Temperature/K**	100.0(4)
**Crystal system**	orthorhombic
**Space group**	*Pccn*
**a/Å**	34.637(2)
**b/Å**	10.3659(6)
**c/Å**	7.8482(5)
**α/°**	90
**β/°**	90
**γ/°**	90
**Volume/Å^3**	2817.9(3)
**Z**	8
**ρ_{calc}g/cm^3**	1.284
**μ/mm^{-1}**	0.085
**F(000)**	1168.0
**Crystal size/mm^3**	0.93 × 0.36 × 0.14
**Radiation**	Mo Kα (λ = 0.71073)
**2Θ range for data collection/°**	4.102 to 49.976
**Index ranges**	$-41 \leq h \leq 40$, $-12 \leq k \leq 12$, $-9 \leq l \leq 9$
**Reflections collected**	24447
**Independent reflections**	2490 [R_{int} = 0.0952, R_{sigma} = 0.0395]
**Data/restraints/parameters**	2490/0/182
**Goodness-of-fit on F^2**	1.126
**Final R indexes [I>=2σ (I)]**	R_1 = 0.1287, wR_2 = 0.3431
**Final R indexes [all data]**	R_1 = 0.1341, wR_2 = 0.3455
**CDCC Identification Code**	2343563

Table S4. Crystallographic parameters for 2(**dimet-ada**)·(**azop**)

**Compound name**	2(**dimet-ada**)·(**azop**)
**Empirical formula**	$C_{18}H_{24}N_2O_2$
**Formula weight**	300.39
**Temperature/K**	293
**Crystal system**	triclinic
**Space group**	*P*-1
**a/Å**	6.8804(4)
**b/Å**	7.3907(5)
**c/Å**	17.5102(9)
**α/°**	89.247(5)
**β/°**	81.049(5)
**γ/°**	73.959(5)
**Volume/Å^3**	844.90(9)
**Z**	2
**ρ_{calc}g/cm^3**	1.181
**μ/mm^{-1}**	0.077
**F(000)**	324.0
**Crystal size/mm^3**	1 × 0.632 × 0.521
**Radiation**	Mo Kα (λ = 0.71073)
**2Θ range for data collection/°**	4.712 to 49.998
**Index ranges**	$-8 \le h \le 8$, $-8 \le k \le 8$, $-20 \le l \le 20$
**Reflections collected**	9515
**Independent reflections**	2952 [R_{int} = 0.0338, R_{sigma} = 0.0259]
**Data/restraints/parameters**	2952/0/202
**Goodness-of-fit on F^2**	1.026
**Final R indexes [I>=2σ (I)]**	R_1 = 0.0640, wR_2 = 0.1621
**Final R indexes [all data]**	R_1 = 0.0818, wR_2 = 0.1725
**CDCC Identification Code**	2343561

Table S5. Crystallographic parameters for 2(**trimet-ada**)·(**azo**)

**Compound name**	2(**trimet-ada**)·(**azop**)
**Empirical formula**	$C_{38}H_{52}N_4O_4$
**Formula weight**	628.83
**Temperature/K**	100.00(10)
**Crystal system**	triclinic
**Space group**	*P*-1
**a/Å**	8.6283(5)
**b/Å**	12.0808(8)
**c/Å**	17.2858(11)
**α/°**	83.226(6)
**β/°**	89.699(5)
**γ/°**	86.075(5)
**Volume/Å^3**	1785.0(2)
**Z**	2
**ρ_{calc}g/cm^3**	1.170
**μ/mm^{-1}**	0.076
**F(000)**	680.0
**Crystal size/mm^3**	1 × 0.56 × 0.04
**Radiation**	Mo Kα (λ = 0.71073)
**2Θ range for data collection/°**	3.912 to 61.322
**Index ranges**	$-12 \leq h \leq 12$, $-17 \leq k \leq 17$, $-24 \leq l \leq 24$
**Reflections collected**	28870
**Independent reflections**	10228 [R_{int} = 0.0575, R_{sigma} = 0.0744]
**Data/restraints/parameters**	10228/0/423
**Goodness-of-fit on F^2**	1.051
**Final R indexes [I>=2σ (I)]**	R_1 = 0.0713, wR_2 = 0.1821
**Final R indexes [all data]**	R_1 = 0.1285, wR_2 = 0.2178
**CDCC Identification Code**	2343566

Table S6. Crystallographic parameters for 2(**adp**)·(**azop**)

**Compound name**	2(**adp**)·(**azop**)
**Empirical formula**	$C_{66}H_{64}N_4O_6$
**Formula weight**	1009.21
**Temperature/K**	100(2)
**Crystal system**	orthorhombic
**Space group**	$Pca2_1$
**a/Å**	61.120(6)
**b/Å**	8.6137(8)
**c/Å**	9.9256(10)
**α/°**	90
**β/°**	90
**γ/°**	90
**Volume/Å^3**	5225.5(9)
**Z**	4
**ρ_{calc}g/cm^3**	1.283
**μ/mm^{-1}**	0.086
**F(000)**	2144.0
**Crystal size/mm^3**	0.07 × 0.03 × 0.005
**Radiation**	synchrotron (λ = 0.7288)
**2Θ range for data collection/°**	4.1 to 46.292
**Index ranges**	$-65 \leq h \leq 65$, $-9 \leq k \leq 9$, $-10 \leq l \leq 10$
**Reflections collected**	54032
**Independent reflections**	6801 [R_{int} = 0.0764, R_{sigma} = 0.0519]
**Data/restraints/parameters**	6801/25/689
**Goodness-of-fit on F^2**	1.088
**Final R indexes [I>=2σ (I)]**	R_1 = 0.0711, wR_2 = 0.1689
**Final R indexes [all data]**	R_1 = 0.0866, wR_2 = 0.1780
**CDCC Identification Code**	2343565

Table S7. Crystallographic parameters for 2(**adp**)·(**bpeta**)

**Compound name**	2(**adp**)·(**bpeta**)
**Empirical formula**	$C_{68}H_{68}N_2O_6$
**Formula weight**	1009.24
**Temperature/K**	100(2)
**Crystal system**	monoclinic
**Space group**	$P2_1/c$
**a/Å**	27.554(3)
**b/Å**	7.3512(8)
**c/Å**	13.0384(14)
**α/°**	90
**β/°**	93.302(4)
**γ/°**	90
**Volume/Å^3**	2636.6(5)
**Z**	2
**ρ_{calc}g/cm^3**	1.271
**μ/mm^{-1}**	0.084
**F(000)**	1076.0
**Crystal size/mm^3**	0.12 × 0.07 × 0.005
**Radiation**	synchrotron (λ = 0.7288)
**2Θ range for data collection/°**	3.036 to 47.906
**Index ranges**	$-30 \leq h \leq 30$, $0 \leq k \leq 8$, $0 \leq l \leq 14$
**Reflections collected**	20257
**Independent reflections**	3417 [R_{int} = 0.0654, R_{sigma} = 0.0555]
**Data/restraints/parameters**	3417/0/347
**Goodness-of-fit on F^2**	1.133
**Final R indexes [I>=2σ (I)]**	R_1 = 0.0793, wR_2 = 0.2297
**Final R indexes [all data]**	R_1 = 0.0970, wR_2 = 0.2459
**CDCC Identification Code**	2343560

Table S8. Selected hydrogen bonds and interactions in single component crystals.

crystal/ parameter	*d*(O-H···O) (Å)	*d*(H···H) (Å)	symmetry code
**ada**	2.6415(9)[1]	-	(1-X, -1-Y, 1-Z)
	-	2.519(9)[2]	(-1+X, +Y, +Z)
**dimet-ada**	2.603(3)[3]	-	(1-X, ½+Y, 1-Z)
	2.659(3)[4]	-	(1-X, ½+Y, 1-Z)
	2.659(3)[5]	-	(1-X, ½+Y, 1-Z)
	2.683(3)[6]	-	(1-X, ½+Y, 1-Z)
**trimet-ada**	2.617(2)[8]	-	-
	2.644(2)[9]	-	-
	-	2.545(12)[10]	(1-X, 1-Y, 1-Z)

[1]O1-H1···O2, [2]H4···H7a, [3]O1-H1···O8, [4]O3-H3···O6, [5]°4···H5-O5, [6]O2···H7-O7, [7]HT···H20A, [8]O1-H1···O4, [9]O3-H3···O2 , [10]H16B···H26C.

Table S9. Selected hydrogen bonds and interactions in co-crystals.

crystal/ parameter	*d*(H···H) (Å)	*d*(N···H) (Å)	*d*(C-H ···π) (Å)	*d*(π ···π) (Å)	symmetry code
2(**ada**)·(**azop**)	-	-	-	-	-
	2.283(10)[1]	-	-	-	(+X, 3/2-Y, -1/2+Z)
	-	2.698(6)[2]	-	-	-
	-	-	2.665(6)[3]	-	(+X, +Y, -1+Z)
	-	-	-	3.720(4)[4]	-
2(**dimet-ada**)·(**azop**)	-	-	-	-	-
	2.724(17)[5]	-	-	-	(+X, 1+Y, +Z)
	-	2.712(3)[6]	-	-	-
	-	-	2.899(12)[7]	-	(2-X, 1-Y, 1-Z)
	-	-	-	3.597(2)[8]	-
2(**trimet-ada**)·(**azop**)	2.493(16)[9]	-	-	-	(1-X, 2-Y, 1-Z)
	-	2.683(2)[10]	-	-	-
	-	2.693(2)[11]	-	-	-
	-	-	2.685(9)[12]	-	-
	-	-	-	3.496(19)[13]	-
2(**adp**)·(**azop**)	2.89(2)[14]	-	-	-	(+X, 3/2-Y, 1/2+Z)
	-	2.633(11)[15]	-	-	(1-X, -1/2+Y, 3/2-Z)
	-	2.649(10)[16]	-	-	
	-	-	2.946(4)[17]	-	(+X, -1+Y, +Z)
	-	-	3.650(4) [18]	-	(1-X, -Y, +Z)
	-	-	3.753(4)[19]	-	(1-X, -Y, 1/2+Z)
	-	-	-	3.760(5)[20]	-
	-	-	-	3.923(4)[21]	-
2(**adp**)·(**bpeta**)	2.836(2)[22]	-	-	-	(+X, 1/2-Y, 1/2+Z)
	-	2.566(6)[23]	-	-	-
	-	-	2.704(2)[24]	-	(+X, 3/2-Y, -1/2+Z)
	-	-	2.810(2)[25]	-	(+X, 3/2-Y, -1/2+Z)
	-	-	2.798(2)[26]	-	(+X, 1/2-Y, 1/2+Z)
	-	-	2.918(2)[27]	-	(1-X, 1/2+Y, 1/2-Z)
	-	-	-	-	-

[1]H10···H8B, [2]O1-H1···N1, [3]C11-H11 ···π (centroid: C14, C13, C12, N1, C16, C15), [4]π ···π (centroid to centroid: C14, C13, C12, N1, C16, C15 to C14, C13, C12, N1, C16, C15), [5]H10···H11B, [6]O1-H1···N1, [7]C15-H3B ···π (centroid: C16, C15, C14, N1, C18, C17), [8]π ···π (centroid to centroid: C16, C15, C14, N1, C18, C17 to C16, C15, C14, N1, C18, C17), [9]H10···H8B, [10]O2-H2···N1, [11]O3-H3···N4, [12]C8-H8A ···π (centroid: C21, C20, N4, C24, C23, C22), [13]π ···π (centroid to centroid: C17, C18, C19, N1, C15, C16 to C17, C18, C19, N1, C15, C16), [14]H22···H20C, [15]O2'-H2'···N4, [16]O2-H2···N1, [17]C14-H14 ···π (centroid: C8, C9, C10, C1, C2, C3), [18]C37-H37 ···π (centroid: C35, C34, N4, C38, C37, C36), [19]C7'-H7' ···π (centroid: C8, C9, C10, C1, C2, C3), [20]π ···π (centroid to centroid: C8, C9, C10, C1, C2, C3 to C32, C31, C30, C29, N1, C33), [21]π ···π (centroid to centroid: C32, C31, C30, C29, N1, C33 to C35, C34, N4, C38, C37, C36), [22]H21B···H22B, [23]O2···H1···N1, [24]C13-H13 ···π (centroid: C9, C10, C1, C2, C3, C8), [25]C14-H14 ···π (centroid: C7, C8, C3, C4, C5, C6), [26]C9-H9 ···π (centroid: C16, C15, C14, C13, C12, C17), [27]C34-H34 ···π (centroid: C8, C3, C2, C1, C10, C9).

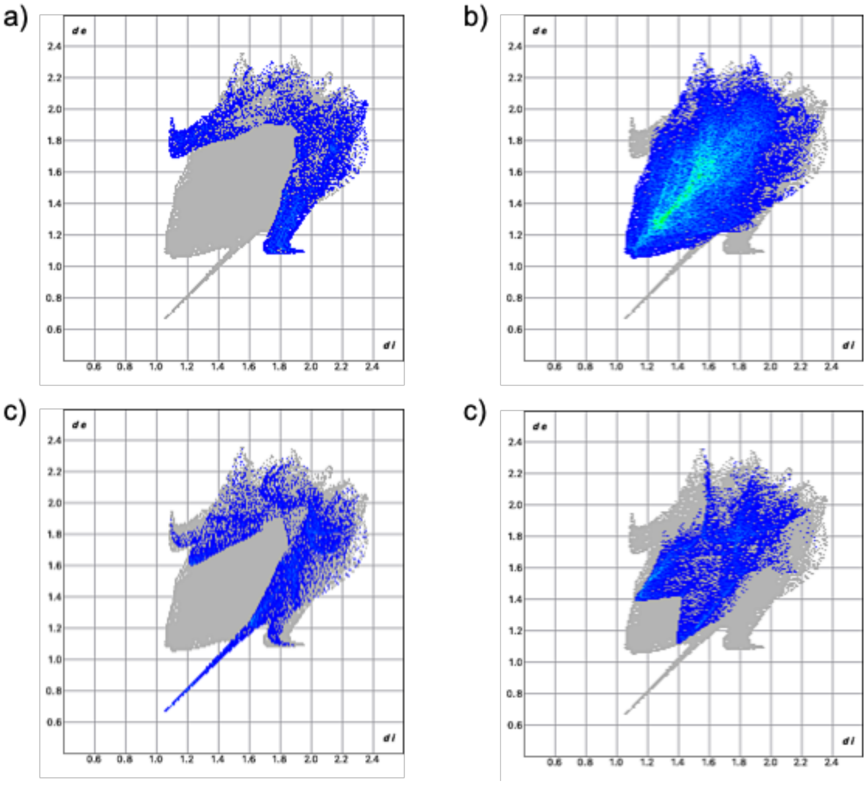

Figure S1. Selected Hirshfeld fingerprint projection interactions for 2(**ada**)·(**azop**) structure: (a) C-H interactions, (b) H-H interactions, (c) N-H interactions, and (d) O-H interactions.

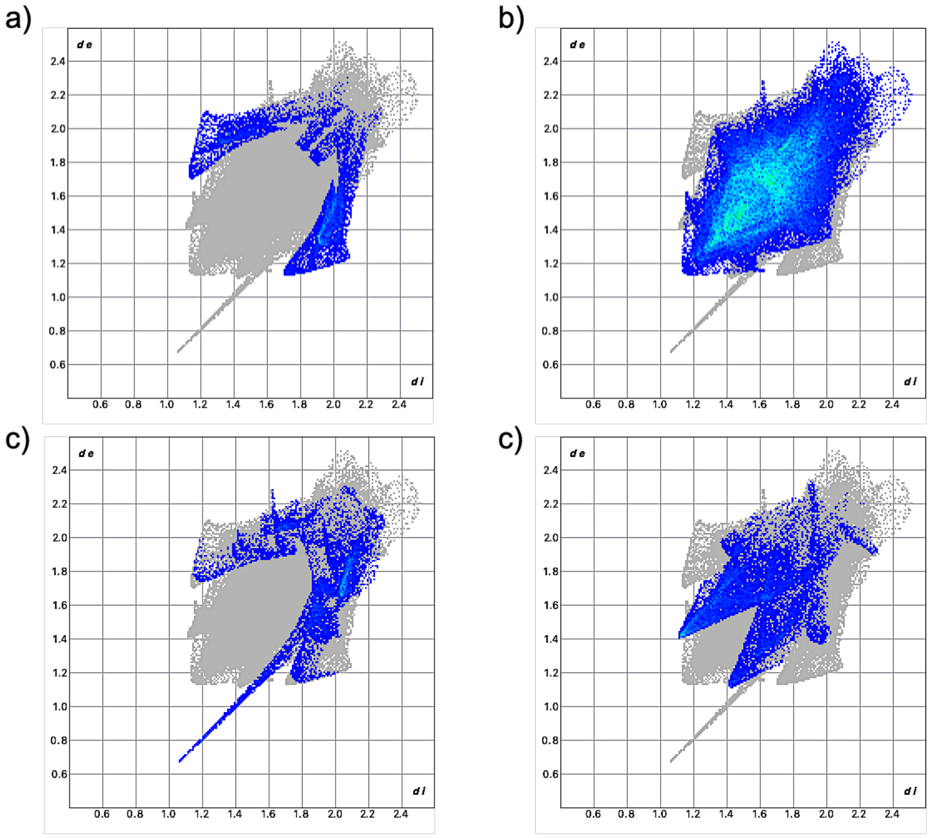

Figure S2. Selected Hirshfeld fingerprint projection interactions for 2(**dimet-ada**)·(**azop**) structure: (a) C-H interactions, (b) H-H interactions, (c) N-H interactions, and (d) O-H interactions.

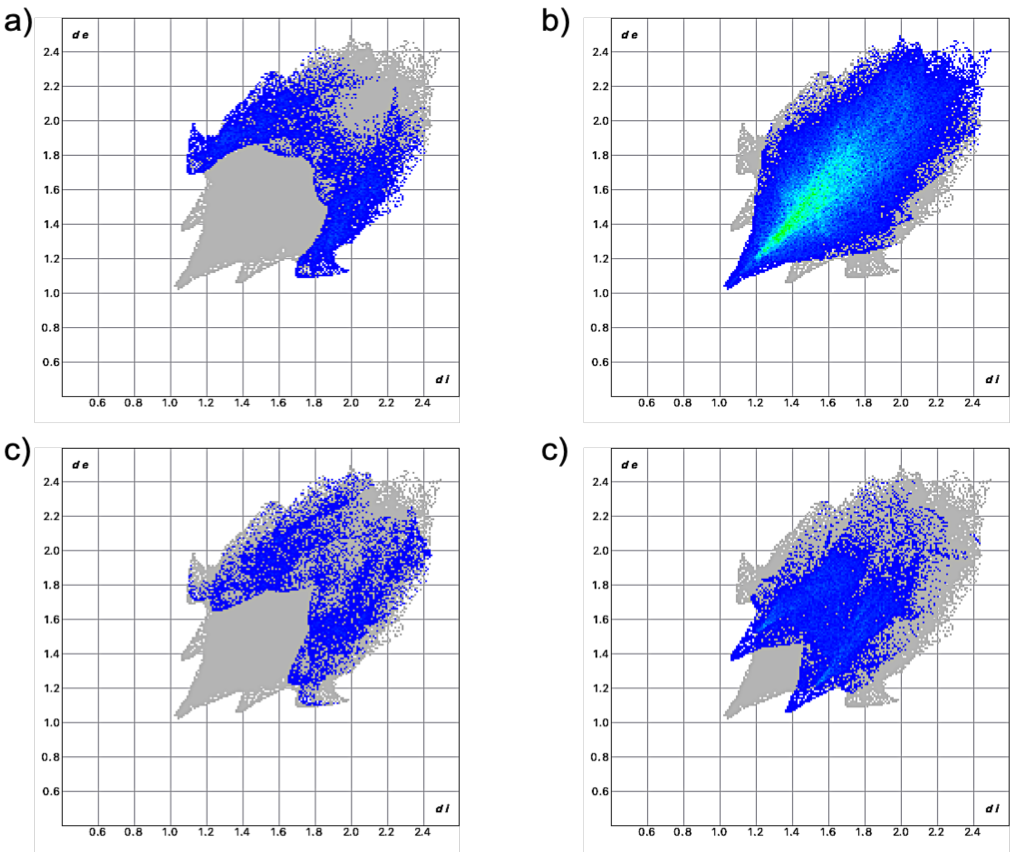

Figure S3. Selected Hirshfeld fingerprint projection interactions for 2(**trimet-ada**)·(**azop**) structure: (a) C-H interactions, (b) H-H interactions, (c) N-H interactions, and (d) O-H interactions.

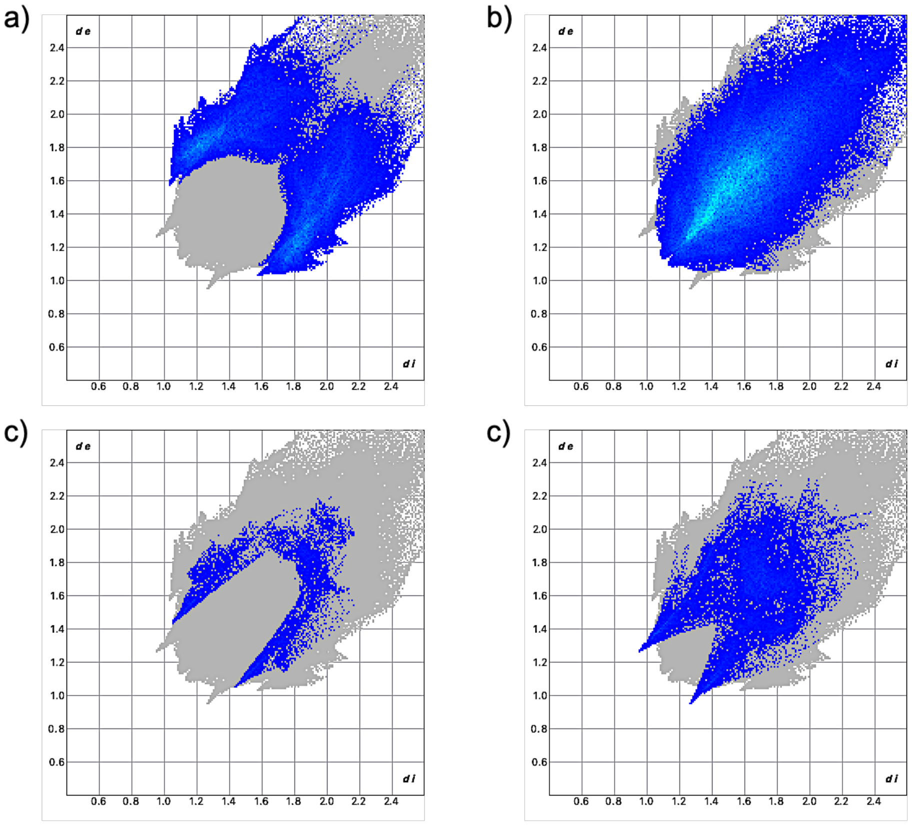

Figure S4. Selected Hirshfeld fingerprint projection interactions for 2(**adp**)·(**azop**) structure: (a) C-H interactions, (b) H-H interactions, (c) N-H interactions, and (d) O-H interactions.

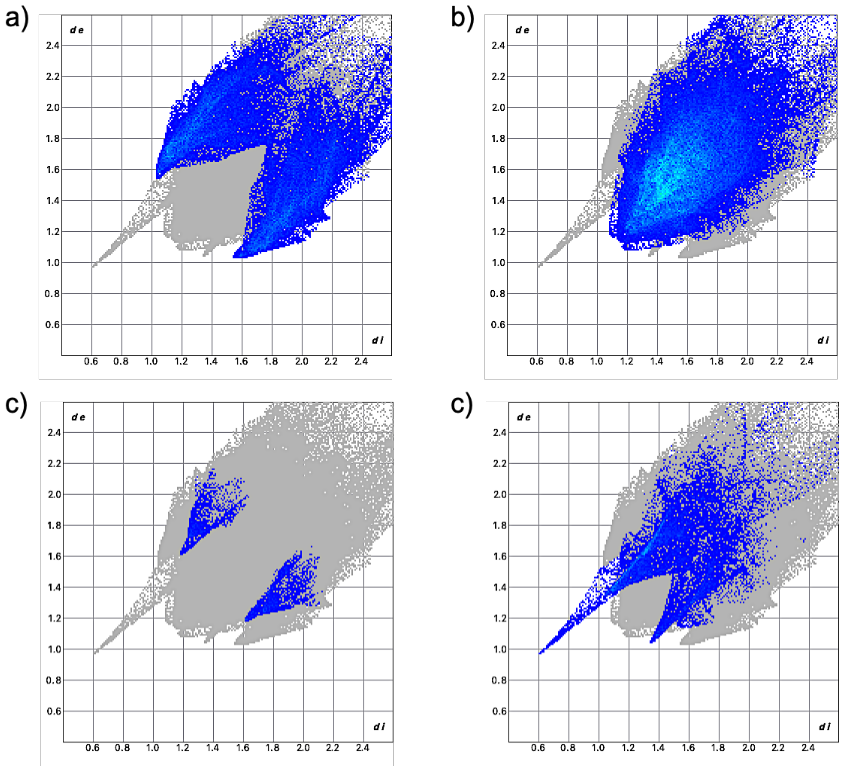

Figure S5. Selected Hirshfeld fingerprint projection interactions for 2(**adp**)·(**bpeta**) structure: (a) C-H interactions, (b) H-H interactions, (c) N-H interactions, and (d) O-H interactions.

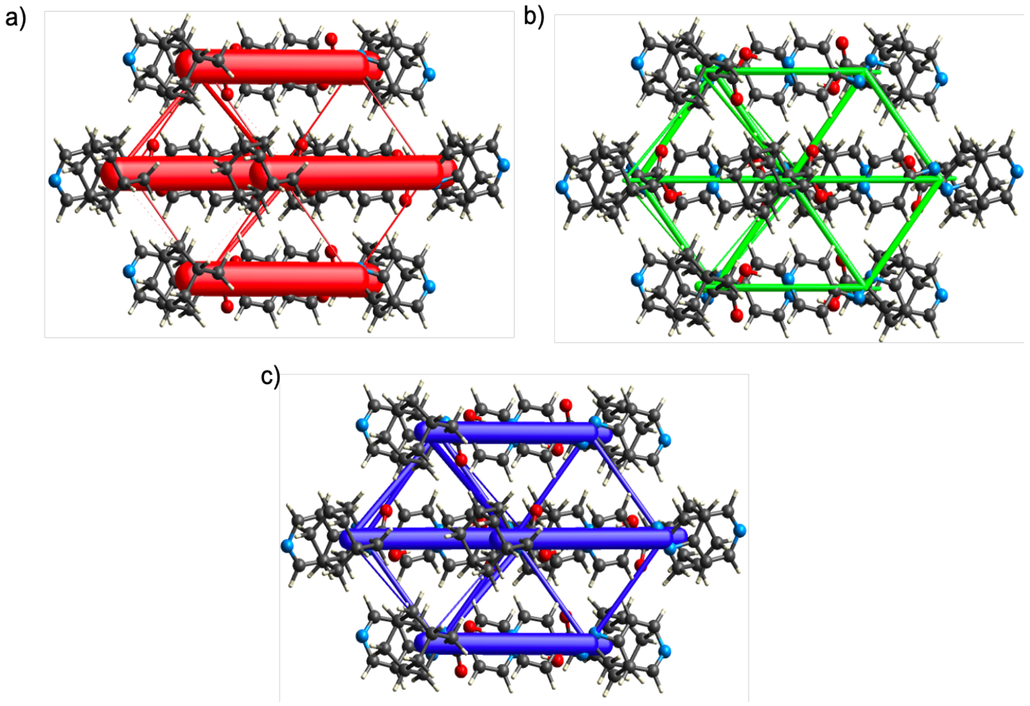

Figure S6. Energy Frameworks along the *a*-axis of 2(**ada**)·(**azop**) calculated using crystal explorer software [HF/3-21G]: a) Coulomb Energy, b) Dispersion Energy, and c) Total Energy.

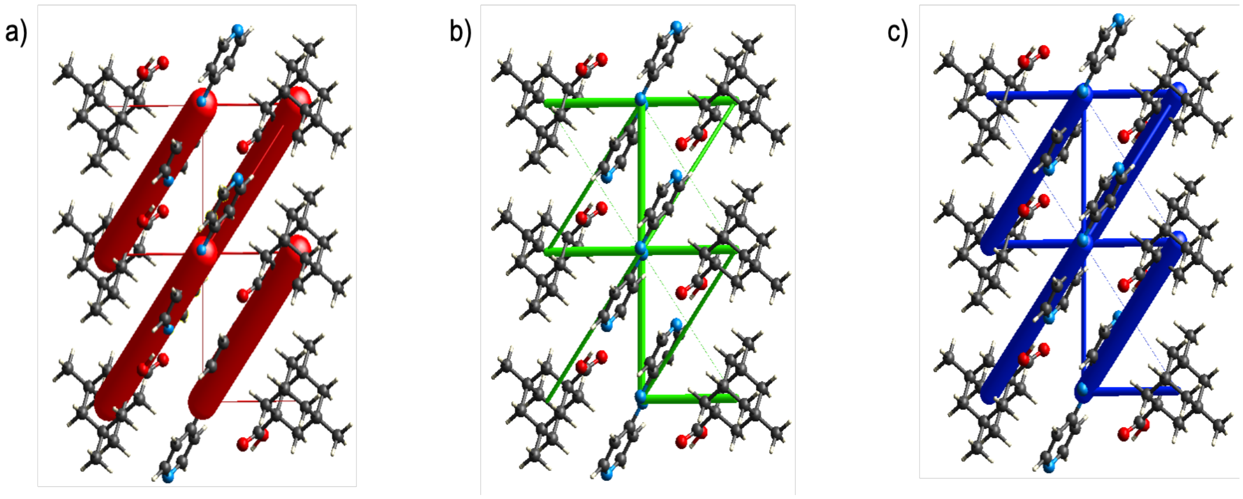

Figure S7. Energy Frameworks along the *a*-axis of 2(**dimet-ada**)·(**azop**) calculated using crystal explorer software [HF/3-21G]: a) Coulomb Energy, b) Dispersion Energy, and c) Total Energy.

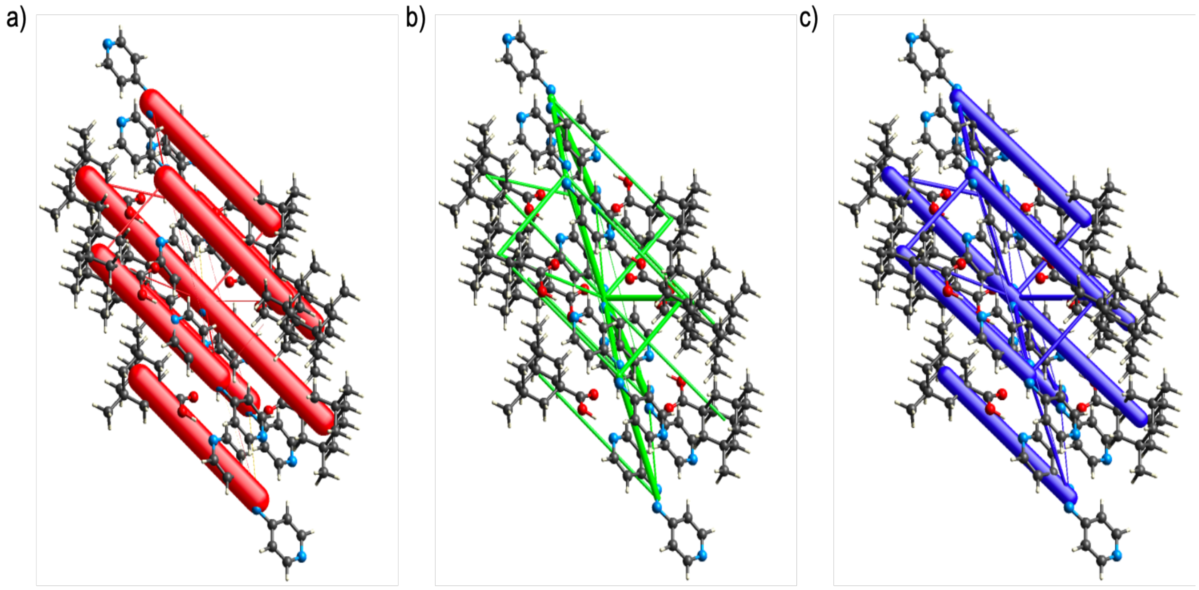

Figure S8. Energy Frameworks along the *a*-axis of 2(**trimet-ada**)·(**azop**) calculated using crystal explorer software [HF/3-21G]: a) Coulomb Energy, b) Dispersion Energy, and c) Total Energy.

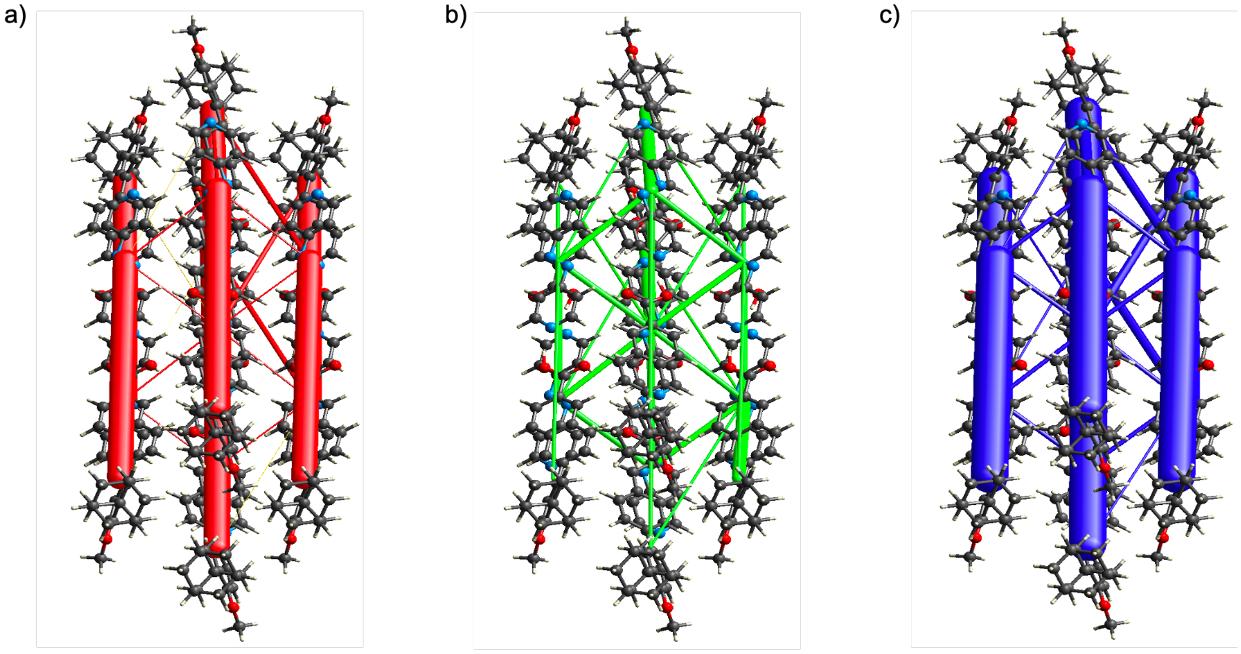

Figure S9. Energy Frameworks along the *a*-axis of 2(**adp**)·(**azop**) calculated using crystal explorer software [HF/3-21G]: a) Coulomb Energy, b) Dispersion Energy, and c) Total Energy.

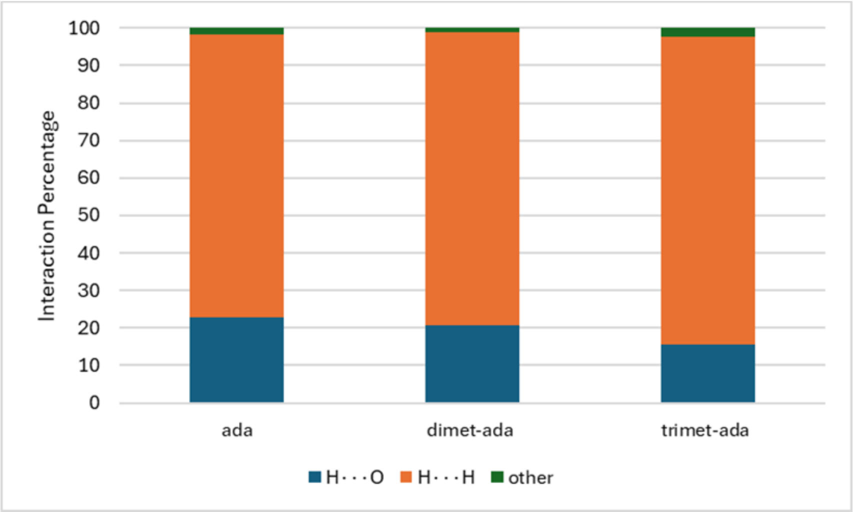

Figure S10. Selected projection interaction percentages of the reported single-component structures.

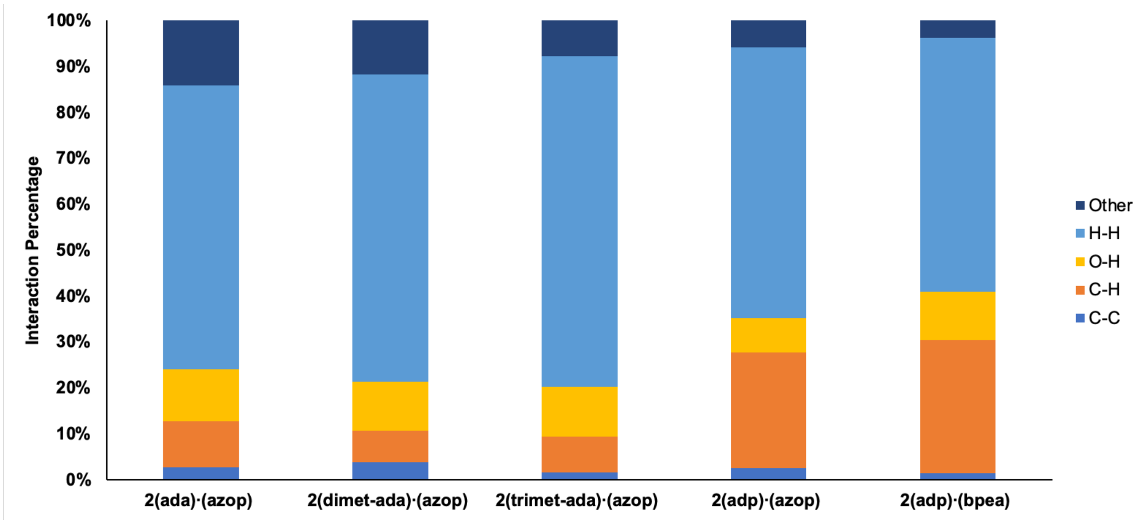

Figure S11. Selected projection interaction percentages of the reported cocrystal structures.

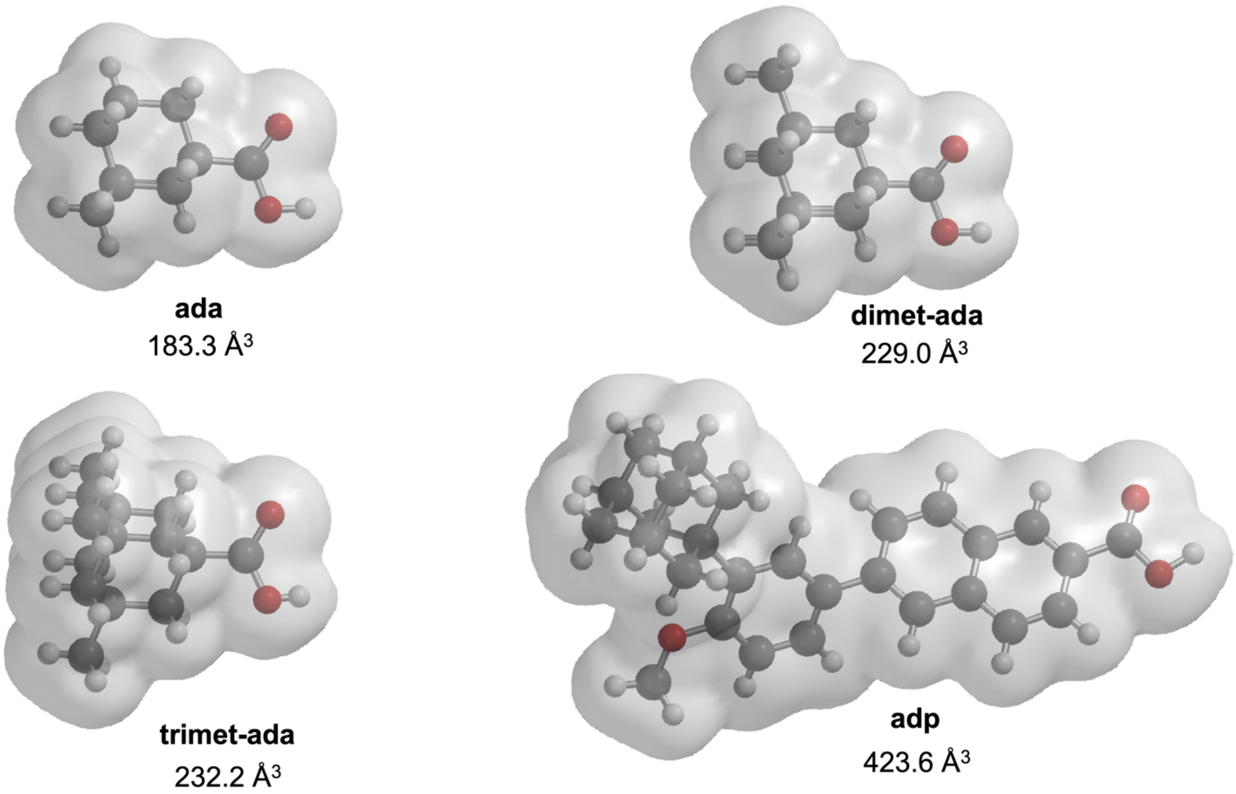

Figure S12. Isodensity surface analysis (0.002 au) of the reported single-component structures. Isodensity surface volumes (Å^3) are shown below the structure.

S3. Powder X-ray diffraction data

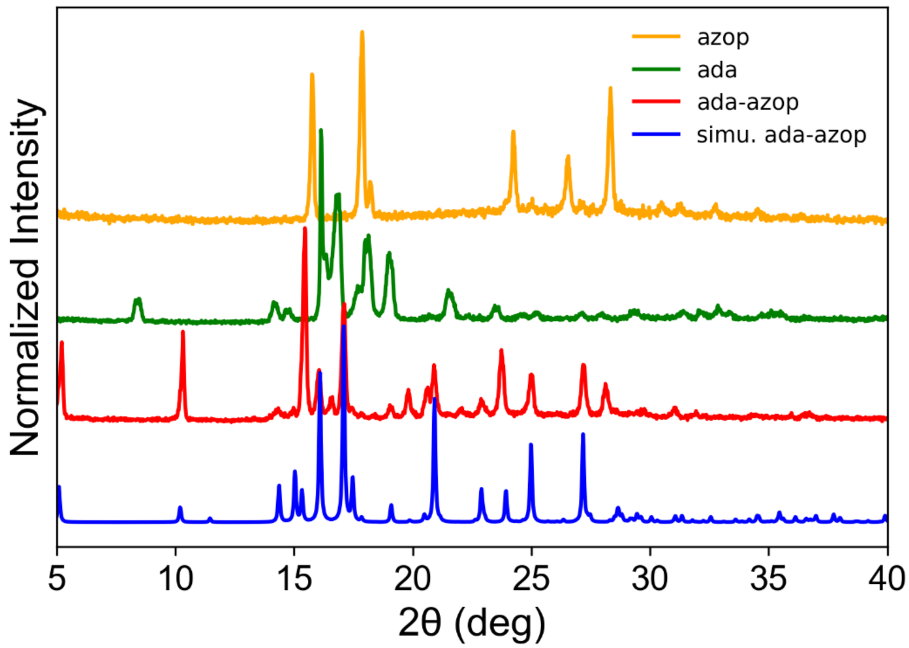

Figure S13. Powder X-ray diffractograms of **azop, ada,** 2(**ada**)·(**azop**), and simulated pattern of 2(**ada**)·(**azop**) from single crystal data.

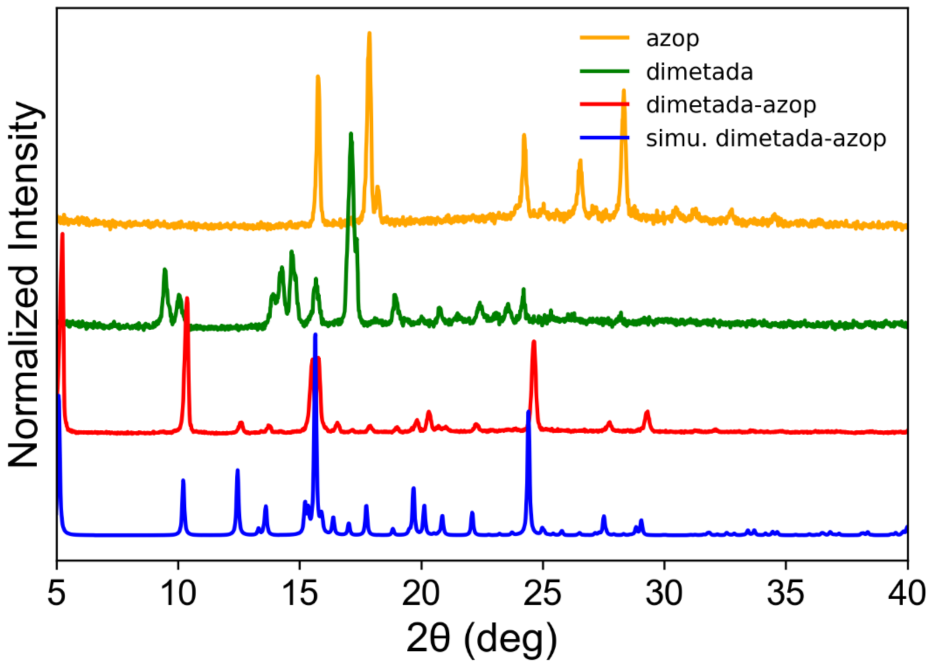

Figure S14. Powder X-ray diffractograms of **azop, dimet-ada,** 2(**dimeta-ada**)·(**azop**), and simulated pattern of 2(**dimet-ada**)·(**azop**) from single crystal data.

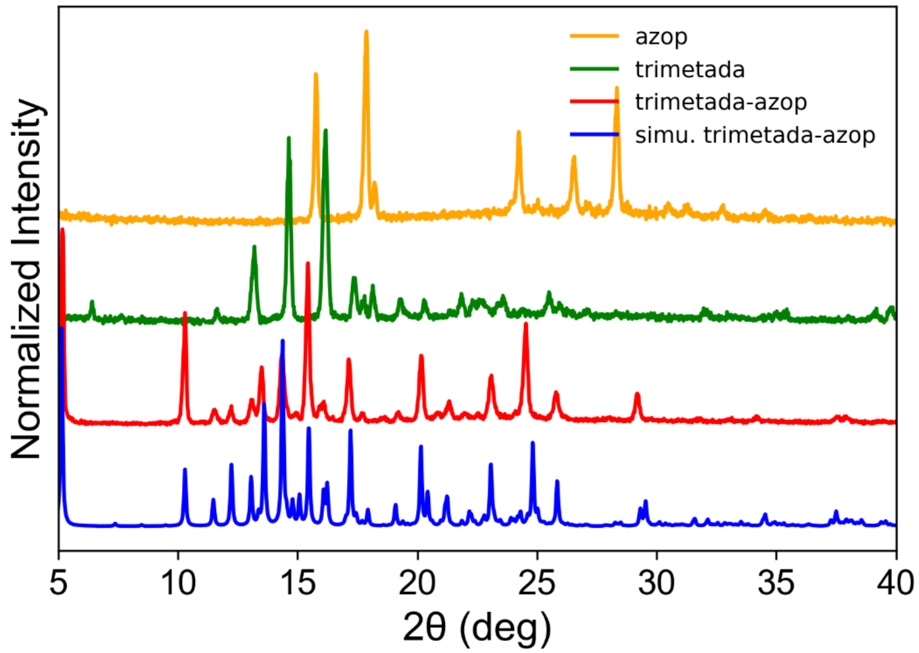

Figure S15. Powder X-ray diffractograms of **azop, trimet-ada,** 2(**trimeta-ada**)·(**azop**), and simulated pattern of 2(**trimet-ada**)·(**azop**) from single crystal data.

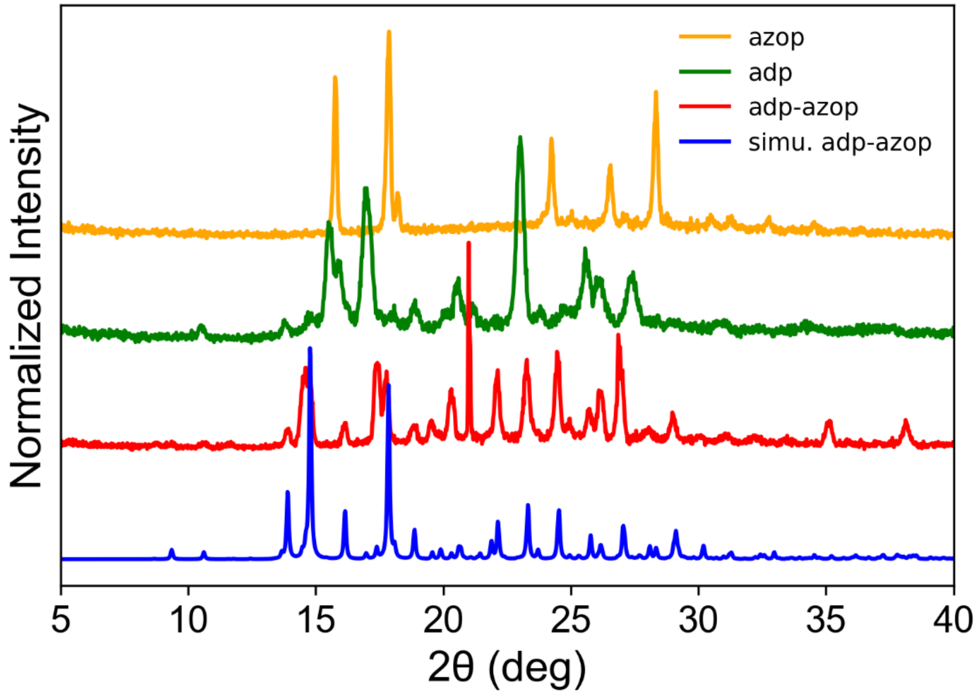

Figure S16. Powder X-ray diffractograms of **azop, adp,** 2(**adp**)·(**azop**), and simulated pattern of 2(**adp**)·(**azop**) from single crystal data.

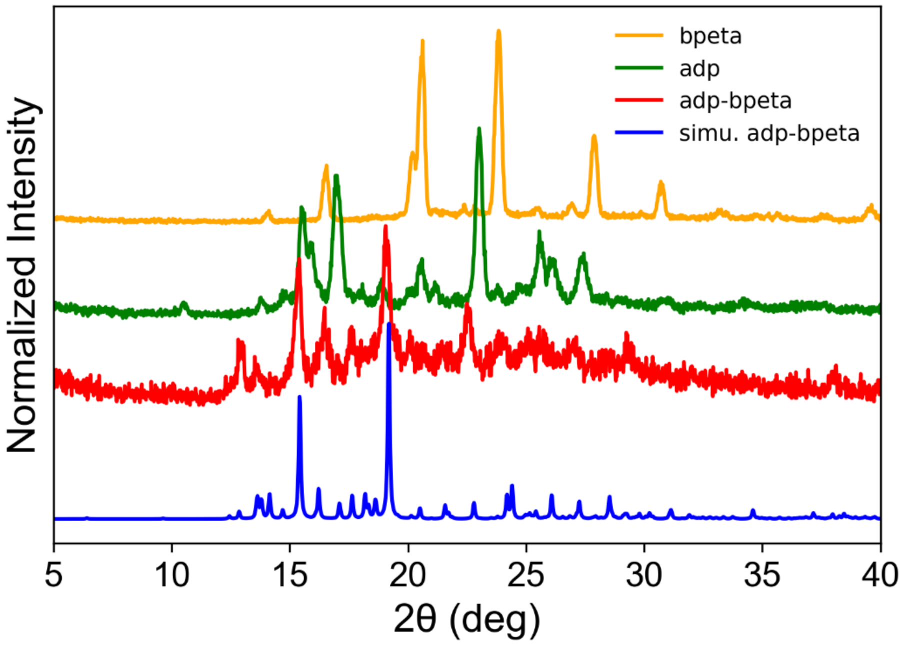

Figure S17. Powder X-ray diffractograms of **bpeta, adp,** 2(**adp**)·(**bpeta**), and simulated pattern of 2(**adp**)·(**bpeta**) from single crystal data.

S5. FT-IR spectral data

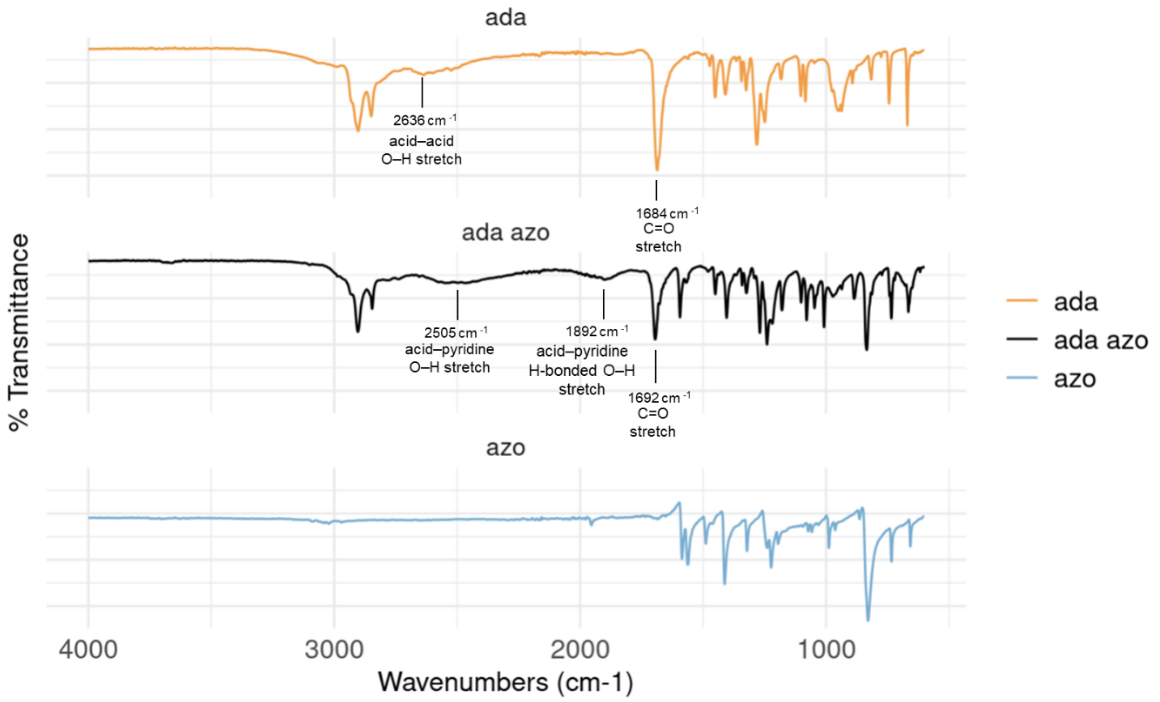

Figure S18. FT-IR spectrum of **ada** and **azop** starting material and their resulting cocrystal, 2(**ada**)·(**azop**). Relevant peaks for cocrystal formation are shown.

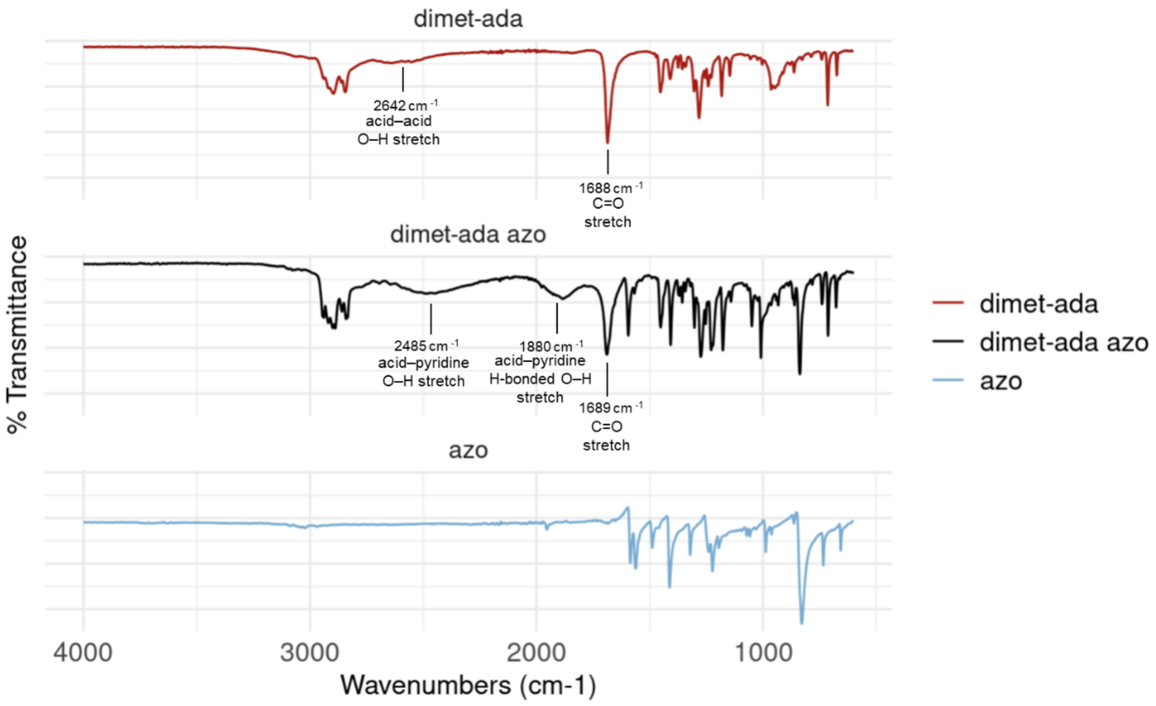

Figure S19. FT-IR spectrum of **dimet-ada** and **azop** starting material and their resulting cocrystal, 2(**dimet-ada**)·(**azop**). Relevant peaks for cocrystal formation are shown.

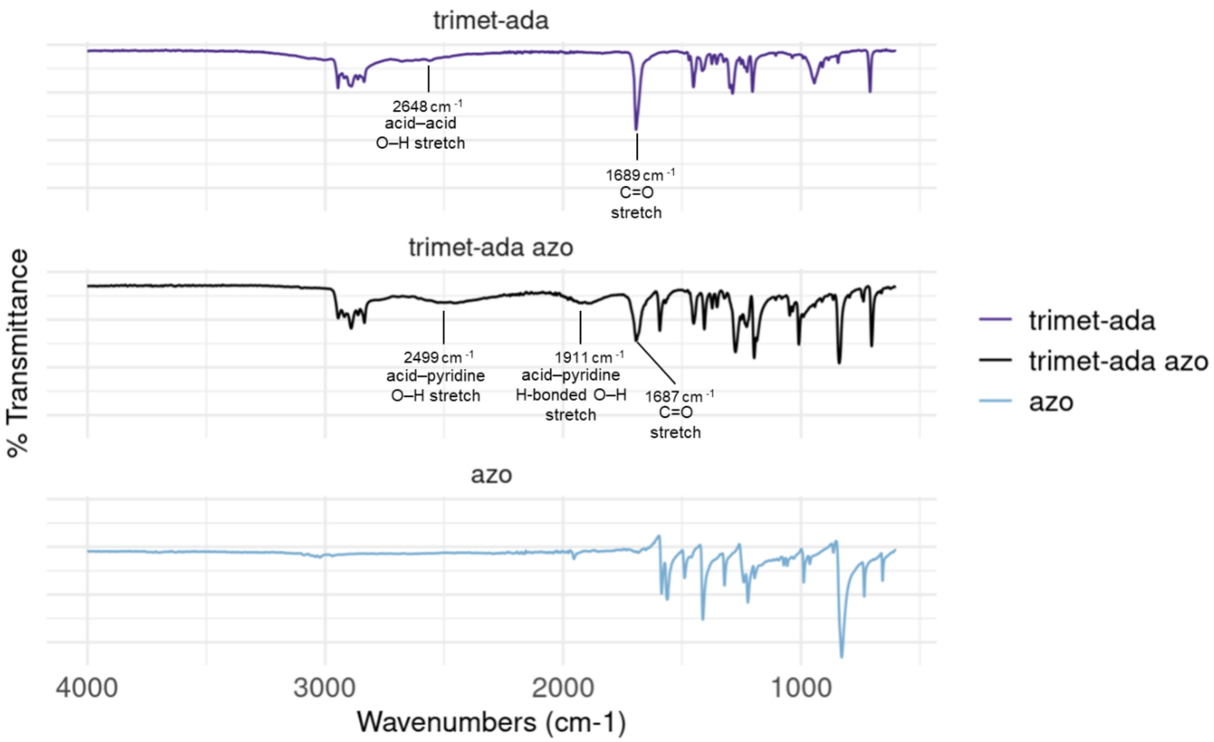

Figure S20. FT-IR spectrum of **trimet-ada** and **azop** starting material and their resulting cocrystal, 2(**trimeta-ada**)·(**azop**). Relevant peaks for cocrystal formation are shown.

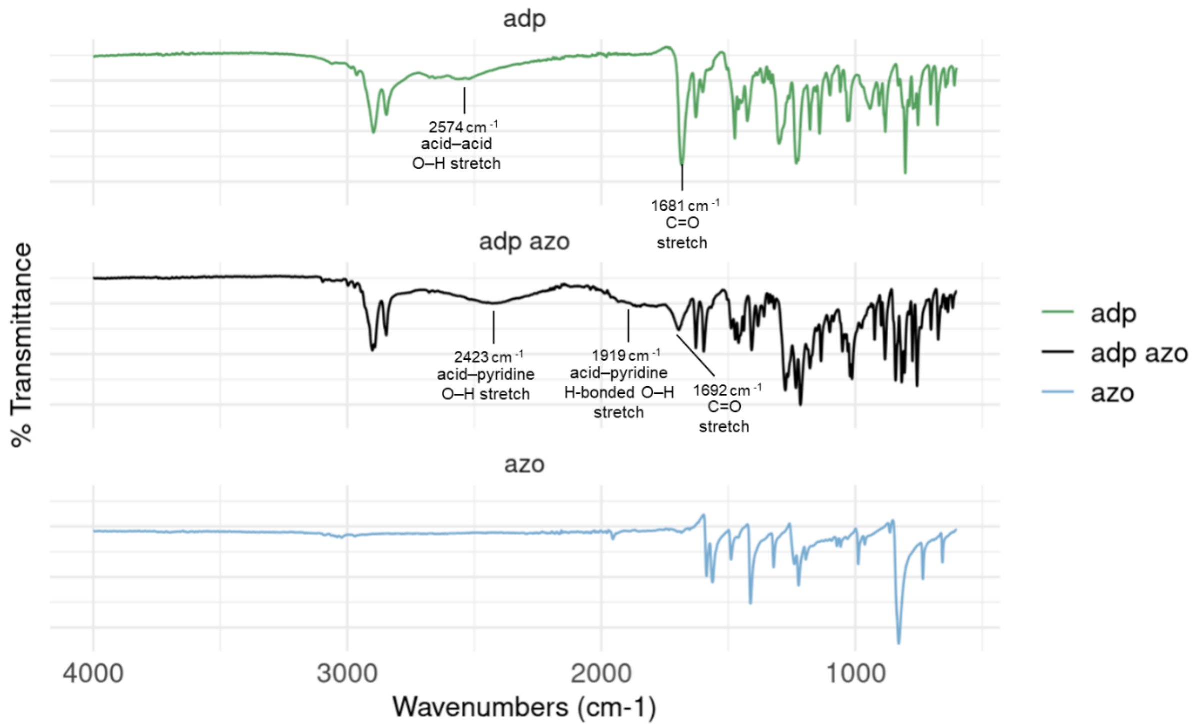

Figure S21. FT-IR spectrum of **adp** and **azop** starting material and their resulting cocrystal, 2(**adp**)·(**azop**). Relevant peaks for cocrystal formation are shown.

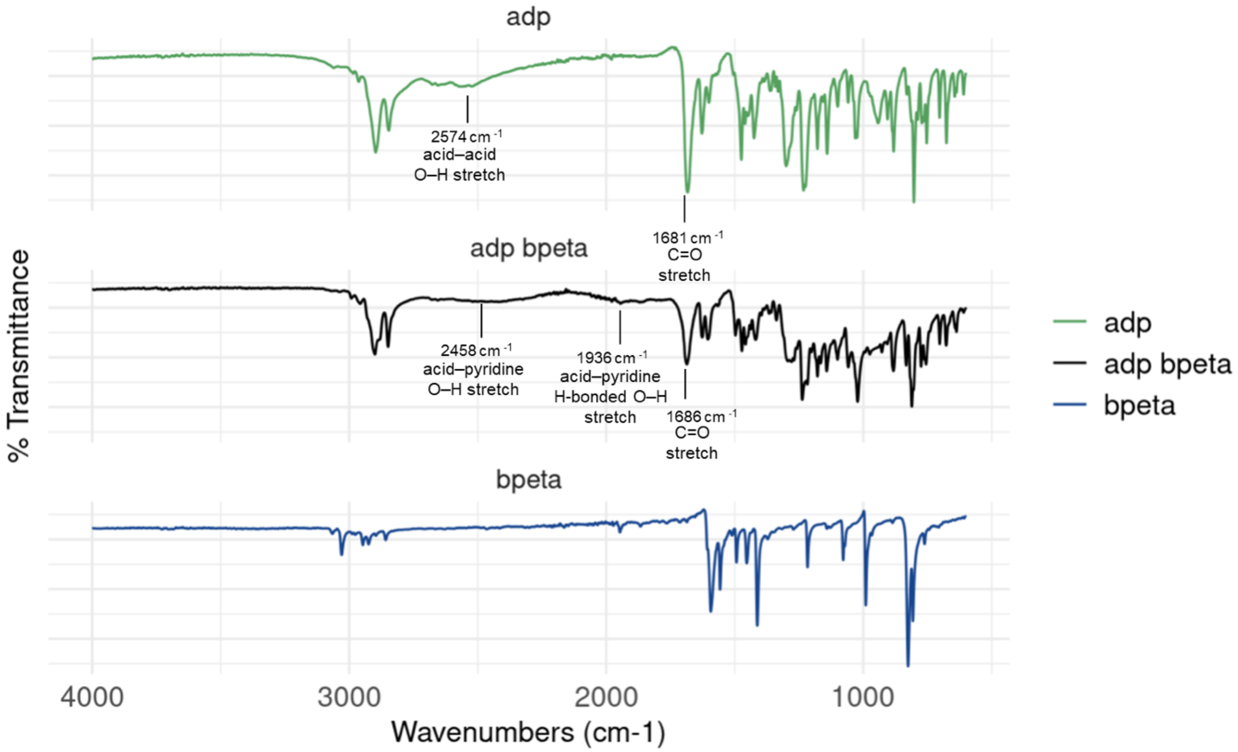

Figure S22. FT-IR spectrum of **adp** and **bpeta** starting material and their resulting cocrystal, 2(**adp**)·(**bpeta**). Relevant peaks for cocrystal formation are shown.

References

(1) Sheldrick, G. M. SHELXT–Integrated space-group and crystal-structure determination. *Acta Cryst. A* **2015**, *71* (1), 3-8.
(2) Sheldrick, G. M. Crystal structure refinement with SHELXL. *Acta Cryst. C* **2015**, *71* (1), 3-8.
(3) Dolomanov, O. V.; Bourhis, L. J.; Gildea, R. J.; Howard, J. A.; Puschmann, H. OLEX2: a complete structure solution, refinement and analysis program. *J. Appl. Crystallogr.* **2009**, *42* (2), 339-341.
(4) Bruker Apex3, Bruker Analytical X-ray Systems Inc., Madison, WI, 2003.
(5) Bruker SAINT: SAX Area-Detector Integration Program v7.60a, Bruker Analytical X-ray Systems, Inc., Madison, WI, 2010.
(6) Blessing, R. *Acta Crystallogr., Sect. A: Found. Crystallogr.* **1995**.
(7) Kissel, L.; Pratt, R. Corrections to tabulated anomalous-scattering factors. *Acta Crystallogr. Sect. A: Found. Crystallogr.* **1990**, *46* (3), 170-175.
(8) Farrugia, L. J. WinGX suite for small-molecule single-crystal crystallography. *J. Appl. Crystallogr.* **1999**, *32* (4), 837-838.
